# Ultrasmall Gold Nanoparticles as “Three-in-One” Enzyme-Mimicking Nanocatalysts for Combined Sonodynamic/Catalytic Therapy in Breast Cancer

**DOI:** 10.1021/acsami.5c15866

**Published:** 2025-12-04

**Authors:** Adilet Beishenaliev, Yean Leng Loke, Chung-Yin Lin, Sook Jing Goh, Jaya Seema, Yuhan Huang, Xin Yun Lim, Yu-Wen Chen, Bey Fen Leo, Chia-Yu Chang, Lip Yong Chung, Chia-Ching Chang, Yin Yin Teo, Dennis W. Hwang, Lik Voon Kiew

**Affiliations:** ‡ Department of Pharmacology, Faculty of Medicine, 37447 Universiti Malaya, 50603 Kuala Lumpur, Malaysia; § Department of Chemistry, Faculty of Science, 37447 Universiti Malaya, 50603 Kuala Lumpur, Malaysia; ¶¶ Research Center for Radiation Medicine, 56081 Chang Gung University, 333 Taoyuan, Taiwan; ∥ Institute of Biomedical Sciences, 38017 Academia Sinica, 11529 Taipei, Taiwan; ⊥ College of Life Sciences, National Yang Ming Chiao Tung University, 30010 Hsinchu, Taiwan; # Biomedical Translation Research Center, 38017 Academia Sinica, 11529 Taipei, Taiwan; ∇ Department of Molecular Medicine, Faculty of Medicine, 37447 Universiti Malaya, 50603 Kuala Lumpur, Malaysia; ○ Nanotechnology and Catalysis Research Centre (NANOCAT), Institute for Advanced Studies, 37447 Universiti Malaya, 50603 Kuala Lumpur, Malaysia; ◆ Universiti Malaya Research Centre for Biopharmaceuticals and Advanced Therapeutics, Faculty of Medicine, 37447 Universiti Malaya, 50603 Kuala Lumpur, Malaysia; ¶ Center of Excellence for Innovative Medical Devices, University Malaya, 50603 Kuala Lumpur, Malaysia; †† Department of Biological Science and Technology, College of Engineering Bioscience, National Yang Ming Chiao Tung University, 30068 Hsinchu, Taiwan; ‡‡ Department of Pharmaceutical Chemistry, Faculty of Pharmacy, 37447 Universiti Malaya, 50603 Kuala Lumpur, Malaysia; §§ Center for Intelligent Drug Systems and Smart Biodevices, National Yang Ming Chiao Tung University, 30068 Hsinchu, Taiwan; ∥∥ Department of Electrophysics, National Yang Ming Chiao Tung University, 30010 Hsinchu, Taiwan; ⊥⊥ Institute of Physics, 38017 Academia Sinica, Nankang, 11529 Taipei, Taiwan; ## Brain Research Center, National Tsing Hua University, 300044 Hsinchu, Taiwan

**Keywords:** ultrasmall gold nanoparticles, ultrasound, sonodynamic therapy, catalytic therapy, sonosensitizer, nanocatalyst

## Abstract

The combination of catalytic therapy and sonodynamic therapy (SDT) emerges as a promising approach for the treatment of solid tumors. Smartly designed nanocatalysts can activate tumor-localized catalytic reactions to convert hydrogen peroxide (H_2_O_2_) into highly toxic hydroxyl radicals (^•^OH) while simultaneously generating singlet oxygen (^1^O_2_) under ultrasound stimulation to induce massive oxidative bursts in cancer cells. However, inadequate levels of endogenous H_2_O_2_ in tumor tissues and poor sonocatalytic performance of existing nanocatalysts remain major challenges for SDT/catalytic therapy. Self-sufficient nanocatalysts that can generate H_2_O_2_ within the tumor microenvironment offer a way to continuously power further catalytic reactions, effectively overcoming the limitations of monomodal nanocatalysts. However, such complex nanocatalyst systems with multienzymatic and sonosensitizing properties are often challenging to translate from the bench to the bedside. In this study, we employed ultrasmall gold nanoparticles (usAuNPs) as “three-in-one” multifunctional yet structurally simple nanocatalysts. Under the mildly acidic environment of tumors, usAuNPs were shown to decompose H_2_O_2_ into ^•^OH via peroxidase-like activity and self-supply H_2_O_2_ by breaking down glucose via glucose oxidase-like activity. Meanwhile, usAuNPs can be activated by ultrasound (1 MHz, 2.0 W/cm^2^) to generate a significant amount of ^1^O_2_ (*k* = 1.78 × 10^–1^ min^–1^), which is 4–7.5-fold greater than other reported gold nanocomposites. *In vivo* experiments showed that usAuNP-mediated SDT/catalytic therapy can cause significant suppression of breast cancer growth, achieving a tumor growth inhibition of 90% following a single dose of nanoparticles. Importantly, their ultrasmall sizes facilitate tumor-specific accumulation and rapid clearance from the body via renal filtration, achieving enhanced cancer eradication without significant systemic toxicity. Overall, this work spotlights a potential use of usAuNPs as effective and simple sonocatalysts for combined SDT/catalytic therapy.

## Graphical abstract

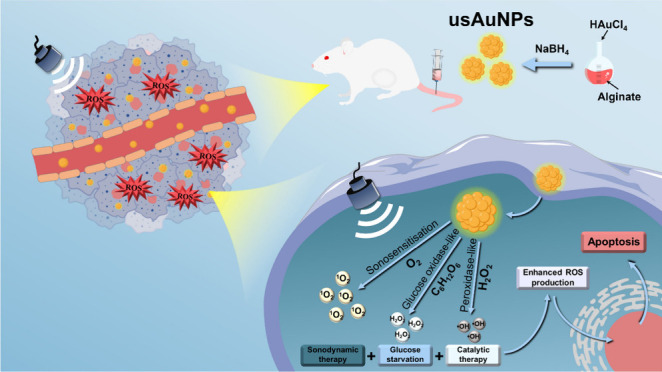

## 1 Introduction

Catalytic therapy using inorganic nanomaterials with enzyme-mimicking activities [e.g., peroxidase (POD), glucose oxidase (GOx), and catalase] has been investigated increasingly for cancer therapy. By initiating a series of catalytic chemical reactions to generate reactive oxygen species (ROS) in tumor tissues, this therapy induces a localized oxidative burst, selectively killing cancer cells with minimal harm to normal tissues/organs.^1^ Combination therapy with other ROS-based cancer treatment modalities [e.g., sonodynamic therapy (SDT),^2^ sonoimmunotherapy,^3,4^ and photodynamic therapy^5^] further enhances therapeutic outcome, providing efficient treatments for solid tumors. However, such approaches are limited by the poor catalytic activity of nanocatalysts and insufficient endogenous substrate molecules [e.g., hydrogen peroxide (H_2_O_2_)] in the tumor tissues.^6^ Thus, developing multifunctional, self-sufficient nanocatalysts with high catalytic efficacy remains a critical challenge.

To address these limitations, various nanocatalyst systems incorporating metals such as Ti,^7^ V,^8^ Mn,^9,10^ Cu,^11^ Pt,^12^ W,^13^ Co,^14,15^ Bi,^16^ Ba,^17,18^ and Au^19^ have been explored for combined SDT/catalytic therapy. These platforms typically integrate a sonosensitizer component [to generate toxic singlet oxygen (^1^O_2_) under ultrasound] with a nanoenzymatic component (to decompose H_2_O_2_ into ^•^OH), thereby synergistically amplifying SDT efficacy.^20^ However, most reported systems are multicomponent nanocomposites that require complex syntheses, which poses challenges in ensuring consistent stability and acceptable biosafety for clinical translation.

In the pursuit of a more streamlined approach, ultrasmall gold nanoparticles (usAuNPs; diameter <5 nm) have attracted interest as single-component nanocatalysts. usAuNPs have been shown to catalyze glucose oxidation, converting glucose and O_2_ into H_2_O_2_ via a mechanism analogous to that of natural GOx enzymes.^21,22^ They also feature intrinsic POD-like activity, rapidly converting H_2_O_2_ into highly cytotoxic ^•^OH radicals.^23–26^ In addition to these enzyme-mimicking functions, usAuNPs are highly stable and biocompatible, and their ultrasmall size allows for rapid renal clearance, thus minimizing potential long-term toxicity from accumulation in organs such as the liver. Notably, we have previously demonstrated that alginate-stabilized usAuNPs can function as effective sonosensitizers, generating substantial amounts of ^1^O_2_ under ultrasound stimulation to kill cancer cells.^27^

Given these properties, we hypothesized that usAuNPs could serve as an all-in-one nanotherapeutic platform to overcome the above challenges. In this study, we investigated the *in vitro* and *in vivo* therapeutic effects of a combined SDT/catalytic therapy treatment mediated by alginate-stabilized usAuNPs (∼3 nm). In the mildly acidic tumor microenvironment, usAuNPs are expected to induce tumor starvation by consuming glucose via GOx-like activity while simultaneously self-supplying H_2_O_2_ to drive ^•^OH generation through POD-like activity. Concurrently, under ultrasound stimulation, usAuNPs generate toxic ^1^O_2_, which can synergistically amplify the overall anticancer effect of the treatment. With these combined capabilities, usAuNPs achieve substantially higher ultrasound-triggered ^1^O_2_ generation compared to several recently reported multicomponent gold-based nanocatalyst systems.^28–32^ This pronounced advantage, together with the particles’ structural simplicity and rapid renal clearance, underscores the potential of usAuNPs as a clinically translatable “three-in-one” agent for synergistic SDT/catalytic cancer therapy.

## 2 Materials and Methods

### 2.1 Materials

Sodium alginate (4–12 cP) and methylene blue (MB) were purchased from Chemiz (Malaysia). Gold­(III) chloride trihydrate (HAuCl_4_·3H_2_O), 1,3-diphenylisobenzofuran (DPBF), 3,3′,5,5′-tetramethylbenzidine (TMB), and fluorescein isothiocyanate isomer I (FITC) were obtained from Sigma-Aldrich (Germany). Sodium borohydride (NaBH_4_) was purchased from Merck (Germany). Hydrogen peroxide (H_2_O_2_) colorimetric detection kit and Annexin V apoptosis detection kit were supplied by Elabscience (USA). Dulbecco’s modified Eagle’s medium (DMEM) and RPMI 1640 were obtained from Nacalai Tesque (Japan). Fetal bovine serum (FBS), Trypsin–ethylenediaminetetraacetic acid (EDTA), phosphate-buffered saline (PBS), and penicillin/streptomycin were purchased from Gibco (USA). 2′,7′-Dichlorodihydrofluorescein diacetate (DCFH-DA) staining kit was obtained from Dojindo Laboratories (Japan). Sodium dodecyl sulfate (SDS) was purchased from Biorad (USA). Primary antibodies (γ-H2AX, Bcl-2, Bax, and β-actin) were supplied by Bioss (USA). M-PERTM mammalian protein extraction reagent was obtained from Abcam (U.K.). All reagents were prepared with Milli-Q ultrapure water unless stated otherwise.

### 2.2 Synthesis of Ultrasmall Alginate-Stabilized usAuNPs

The synthesis procedure and physicochemical characterization of alginate-stabilized usAuNPs were described in detail in our previous work.^27^ Briefly, 5 mL of 1.5 mg/mL NaBH_4_ was added to 160 mL of the mixture containing 0.275 mM HAuCl_4_ and 1 mg/mL sodium alginate under vigorous stirring. The resulting usAuNPs were washed three times with ultrapure water using 100 kDa Viva spin (Sartorius, Germany) at 2700*g* for 15 min and stored at 4 °C until further use. For fluorescence imaging, usAuNPs were labeled with FITC by mixing as-synthesized dispersion of usAuNPs with 50 μM FITC under continuous stirring at room temperature for 24 h. The mixture was then washed three times with ultrapure water at 9000*g* for 10 min, and the resulting usAuNPs-FITC was stored at 4 °C until further use.

### 2.3 Physicochemical Characterization

The optical spectrum was measured using a NanoDrop 2000c spectrophotometer (Thermofisher, U.K.) from 190 to 840 nm. The hydrodynamic diameter and ζ potential of usAuNPs were recorded by using dynamic light scattering (DLS; NanoZS, Malvern Instruments, U.K.). The nanoparticle diameter and structure of usAuNPs were observed by transmission electron microscopy (TEM; JEOL-2100F, JEOL, Japan) operating at a 200 kV accelerating voltage. Energy-dispersive X-ray (EDX) spectroscopy was performed using a Talos F200X G2 S/TEM (Thermo Scientific, USA). X-ray diffraction (XRD) pattern was performed using an EMPYREAN X-ray diffractometer (Malvern Panalytical, U.K.). The stability of usAuNPs was assessed by measuring the change in the hydrodynamic size of usAuNPs (50 μg/mL) dispersed in ultrapure water, complete RPMI 1640, and PBS with 10% FBS at 37 °C for 14 days. After 14 days, the samples were centrifuged using 100 kDa Viva spin (Sartorius, Germany) at 2700*g* for 15 min, freeze-dried for 3 days, and analyzed via Fourier transform infrared (FTIR) spectroscopy (FTIR-Spectrum 400, PerkinElmer, USA) operating in transmittance mode in the 4000–450 cm^–1^ scanning region (15 scans per sample).

### 2.4 Detection of ^•^OH Generation via POD-Like Activity

H_2_O_2_ decomposition via POD-like activity was assessed by incubating 0–200 μg/mL usAuNPs with 100 mM H_2_O_2_ for 24 h in the dark. The H_2_O_2_ concentration was measured with a standard H_2_O_2_ colorimetric assay kit based on ammonium molybdate, which forms a yellow complex with an absorbance peak at 405 nm upon reaction with H_2_O_2_. The absorbance values were recorded using a NanoDrop 2000c spectrophotometer (Thermofisher, U.K.). The generation of ^•^OH following the decomposition of H_2_O_2_ by usAuNPs was also investigated using MB as a probe.^33^ A total of 8 μL of 500 μg/mL MB was mixed with 492 μL of 50 μg/mL usAuNPs containing 0–8 mM H_2_O_2_ and incubated for 120 min. The change in MB absorbance was recorded every 20 min using a NanoDrop 2000c spectrophotometer. The first-order rate constant was calculated as in the equation

1 $$\ln\left(A_{t}/A_{0}\right)=-kt$$

where *A*_0_ and *A*_*t*_ represent absorbance values of MB at the start and after a specific time *t*, respectively, measured at 660 nm. The rate constant *k* was determined by fitting the data to a linear function.

The POD-like activity of usAuNPs was further evaluated using TMB colorimetric assay. In the presence of H_2_O_2_, usAuNPs facilitate the generation of ^•^OH, resulting in the formation of blue-colored oxidized TMB (oxTMB). The reaction mixture was prepared by mixing 100 μL of TMB with 100 μL of usAuNPs (100 μg/mL). H_2_O_2_ (0.5–10 mM final concentration) was added to the mixture, and the solution was incubated for 15 min in the dark. The UV–vis absorbance of each sample was measured every 60 s at 652 nm using a NanoDrop 2000c spectrophotometer. The absorbance values were converted to the concentration of oxTMB in the reaction samples using Beer–Lambert law:

2 A=εlc

where *A* is the absorbance of the reaction solution at 652 nm, ε is the molar extinction coefficient of oxTMB (39000 M^–1^ cm^–1^), *l* is the optical length (1 cm), and *c* is the concentration of oxTMB in the reaction solution. Subsequently, the obtained initial reaction velocity (*V*_0_) values were plotted against the H_2_O_2_ concentration to generate a Michaelis–Menten curve and then transformed to Lineweaver–Burk double-reciprocal plots to determine the maximum reaction velocity (*V*_max_) and Michaelis–Menten constant (*K*_M_) as in the following equation:

3 $$1/V_{0}=K_{M}/V_{\max}\left[H_{2}O_{2}\right]+1/V_{\max}$$

### 2.5 Detection of H_2_O_2_ Generation via GOx-Like Activity

Potassium permanganate (KMnO_4_) was used as a probe to detect the formation of H_2_O_2_ via glucose oxidation.^34–37^ Briefly, 25 μL of 10 mM KMnO_4_ was added to 375 μL of 0–200 μg/mL usAuNPs containing glucose (500 μg/mL) at room temperature. After 20 min, the samples were centrifuged using 100 kDa centrifugal filter units at 10000*g* for 7 min, and the absorbance of the filtrate was measured using a NanoDrop 2000c spectrophotometer (Thermofisher, U.K.). To measure the change in pH, 0.625 mL of 10 mM KMnO_4_ was added to 9.375 mL of 200 μg/mL usAuNPs containing 500 μg/mL glucose. pH measurements were performed every 10 min.

### 2.6 Detection of ^1^O_2_ Generation via Ultrasound Activation

^1^O_2_ generation by usAuNPs under ultrasound irradiation was detected by using a DPBF probe. A total of 250 μL of a mixture containing 50 μg/mL usAuNPs and 50 μM DPBF was added to a 96-well plate and irradiated with ultrasound at 0–2.0 W/cm^2^ (1 MHz, 100% duty cycle) for 5 min in the dark. The absorbance was recorded at 420 nm every 1 min. The first-order rate constant was calculated as in eq 1. Electron spin resonance (ESR) spectroscopy was further performed to detect the generation of ^1^O_2_. A series of mixtures containing 10 mM 2,2,6,6-tetramethyl-4-piperidone (TEMP) and 0–200 μg/mL usAuNPs were irradiated with ultrasound (2.0 W/cm^2^, 100% duty cycle) for 3 min in the dark. After ultrasound irradiation, the samples were analyzed using a ESR spectrometer (Bruker ELEXSYS E580, USA).

### 2.7 Cell Lines

Murine fibroblasts, L929, and murine triple-negative breast cancer cells, 4T1, were cultured in T75 tissue culture flasks in DMEM and RPMI 1640, respectively, each containing 10% FBS and 1% penicillin/streptomycin. The cells were grown in a humidified cell incubator at 37 °C under a 5% CO_2_ atmosphere. The cells were trypsinized at 80% cell confluence using Trypsin–EDTA, seeded in the plates after counting, and allowed to grow overnight before treatment protocols were applied.

### 2.8 *In Vitro* Biocompatibility

Cytocompatibility of usAuNPs was examined by using the L929 cell line. The cells were seeded in a 96-well plate at 5000 cells/well and allowed to grow overnight. The cells were treated in triplicate with usAuNPs at various concentrations, ranging from 0 to 200 μg/mL. The cell viability was assessed via MTT assay after 24 h. The absorbance values were measured at 570 nm using a microplate reader (Plate CHAMELEON V, Hidex Oy, Finland).

The hemocompatibility of usAuNPs was assessed on murine red blood cells (RBCs). A total of 200 μL of usAuNPs (0–200 μg/mL in PBS) was mixed with 200 μL of a 4% RBCs suspension. Triton X-100 [1% (v/v)] and PBS solutions were mixed with 4% RBCs to prepare positive and negative controls, respectively. Following a 5 h incubation period, samples were centrifuged (1000*g* for 10 min at 4 °C), and supernatants were transferred to a 96-well plate for absorbance measurements of released hemoglobin (λ = 570 nm) using a microplate reader. The degree of hemolysis was calculated using the following equation:

4 $$\mathrm{hemolysis}\;\%=[\left(A_{\mathrm{sample}}-A_{\mathrm{NC}}\right)/\left(A_{\mathrm{NC}}-A_{\mathrm{PC}}\right)]\times 100$$

where *A*_sample_, *A*_NC_, and *A*_PC_ are absorbance measurements of the nanoparticle samples, negative control, and positive control, respectively.

### 2.9 *In Vitro* Intracellular ROS Detection

DCFH-DA staining assay was used to investigate intracellular ROS production under ultrasound by usAuNPs. Briefly, 4T1 cells were seeded in a 96-well plate (4000 cells/well) and grown overnight. Then, the cells were incubated with RPMI 1640 media containing 10 μM DCFH-DA. After 30 min, the cells were washed with PBS twice and then treated with usAuNPs (100 μg/mL) for an additional 1 h. For the SDT group, the cells were irradiated with ultrasound (2.0 W/cm^2^, 100% duty cycle) for 2 min. The cells were observed under an inverted fluorescence microscope (Eclipse TI-S, Nikon, Japan).

### 2.10 *In Vitro* Catalytic Therapy and SDT

The anticancer catalytic and sonotoxic effects of usAuNPs were investigated on 4T1. The cells were seeded in 96-well plates at 4000 cells/well and grown overnight. The cells were treated with RPMI 1640 media, containing various concentrations of usAuNPs, ranging from 0–200 μg/mL, and incubated for 24 h to observe the catalytic activity of usAuNPs. For SDT, the cells were irradiated with ultrasound (1 MHz, 2.0 W/cm^2^, 100% duty cycle) for 2 min (one well at a time) after 1 h of incubation. A therapeutic ultrasound machine (BTL-4710, BTL Industries, U.K.) equipped with a probe with a surface area of 1 cm^2^ was used. The ultrasound probe was coated with a thin layer of gel and positioned underneath the wells containing cells. The cell viability was evaluated after 24 h via MTT assay.

The cell apoptosis was detected via flow cytometry based on Annexin V-FITC and propidium iodide staining. The 4T1 cells were treated with 100 μg/mL usAuNPs and irradiated with ultrasound following the aforementioned procedure. The cells were stained after 6 h and analyzed using a flow cytometer (BD FACSCanto II, BD Biosciences, USA) based on 10000 events.

### 2.11 Western Blot

Western blotting was performed to investigate the expression of apoptosis-associated proteins in 4T1 cells after combined SDT/catalytic therapy of usAuNPs. The cells (2 × 10^5^) were seeded in 10 cm culture dishes and grown overnight. 4T1 were treated with 100 μg/mL usAuNPs for 1 h and then irradiated with ultrasound (2.0 W/cm^2^, 2 min). After 24 h, the cells were lysed with a M-PERTM mammalian protein extraction reagent with a protease/phosphatase inhibitor for 30 min on ice and then collected at 10000*g* for 10 min at 4 °C. Equal portions of lysate (15–50 μg total protein per lane) were added to 12% SDS–polyacrylamide gel and separated via electrophoresis. The proteins were then transferred to a poly­(vinylidene difluoride) membrane (0.45 μm) and immunoblotted using primary antibodies (β-actin, Bcl-2, Bax, and γ-H2AX) after the membrane was blocked using 5% bovine serum albumin. Finally, the membranes were rinsed with Tris-buffered saline containing 0.1% Tween-20 and immunoblotted with horseradish peroxidase (HRP)-conjugated secondary antibodies. The corresponding bands were detected using a chemiluminescent HRP substrate and captured via an ImageQuant LAS4000 chemiluminescence system (GE HealthCare, USA).

### 2.12 Animal Model

Female wild-type BALB/c mice (15–20 g; 6–8 weeks old) were used for *in vivo* experiments. For acute toxicity and biodistribution studies, animals were acquired from BioLASCO (Taiwan) and kept in the Animal Room of Animal Imaging Facility, Biomedical Translation Research Center (BioTReC), Academia Sinica, Taiwan. For the anticancer efficacy study, animals were acquired from the Comparative Medicine and Technology Unit, Universiti Putra Malaysia (Malaysia), and kept in the Association for Assessment and Accreditation of Laboratory Animal Care International-Accredited Animal Facility at the Animal Experimental Unit, Faculty of Medicine, Universiti Malaya, Malaysia. All animals were maintained at a standardized humidity of 65 ± 5%, a temperature of 22 ± 1 °C, and a 12:12 light–dark cycle, with continuous access to rodent chow and tap water *ad libitum* throughout the study. All animal studies were conducted in compliance with guidelines approved by the Institutional Animal Care and Use Committee from Academia Sinica (Ethics Reference No. BioTReC-110-M-015-R7) and Universiti Malaya (Ethics Reference No. 2023-260228/PHARM/R/AB).

### 2.13 *In Vivo* Toxicity

The toxicity profile of usAuNPs was assessed following the intravenous injection of nanoparticles at 0–16 mg/kg dosages (prepared in PBS) to healthy female BALB/c mice (*n* = 3) via tail vein. The mice were monitored for typical symptoms of toxicity such as body weight loss, inactivity, ruffled fur, and behavior changes.^38^ At the end of the monitoring period, the mice were sacrificed, and major organs (liver, spleen, kidneys, heart, and lungs) were sampled for histopathological analysis.

### 2.14 *In Vivo* Biodistribution

A suspension of 4T1 murine breast cancer cells at a density of 2 × 10^6^ in 100 μL of PBS was injected orthotopically into the mammary fat pads of the mice. Mice were used in all subsequent studies once the tumor volume reached 100 mm^3^. The tumor volume was measured using a calliper and estimated using the following equation:

5 $$\mathrm{tumor volume}\;\left({\mathrm{mm}}^{3}\right)=W^{2}L/2$$

where *W* is the shortest dimension and *L* is the longest dimension.^26^ Tumor-bearing mice (*n* = 6) were injected intravenously with a single dose of 16 mg/kg usAuNPs via tail vein. Mice were sacrificed at specific time points (5 min, 1 h, 2 h, 4 h, 12 h, 24 h, and 6 days after injection), and biological samples including kidney, liver, tumor, blood, and urine were collected, weighed, and stored at −20 °C. Samples were digested in freshly prepared aqua regia [4:1 (v/v) ratio of 37% (w/v) HCl and 64% (w/v) HNO_3_] and incubated at 90 °C for 12 h. The digested samples were then diluted in ultrapure water and centrifuged at 1000*g* for 10 min to remove tissue debris. The supernatants were analyzed by inductively coupled plasma mass spectrometry (ICP-MS; Shimadzu ICPMS 2040, Japan) to measure the Au content. A standard calibration curve was obtained using a standard solution of ionic Au.

### 2.15 *In Vivo* Anticancer SDT/Catalytic Therapy

The anticancer efficacy of usAuNPs as nanocatalysts was investigated by injecting tumor-bearing mice (*n* = 6) with a single dose of 16 mg/kg usAuNPs intravenously via the tail vein. At 4 h postinjection, tumors were irradiated with ultrasound (1 MHz, 2.0 W/cm^2^, 100% duty cycle) for 5 min. The ultrasound probe was coated with a thin layer of gel to ensure proper ultrasound conductivity and positioned directly against the tumor. The intensity and duration of ultrasound were optimized to ensure no damage from direct ultrasound treatment itself without any nanoparticle administration. The tumor temperature was recorded using a MET-FLTG300+2 thermal camera (SEAT Industry Technology, Taiwan). The tumor volume changes were recorded using a calliper every 2 days for 14 days and calculated as in eq 5. On day 14, the tumor growth inhibition (TGI) was calculated using the following equation:

6 TGI%=[1−(ΔT/ΔC)]×100

where Δ*T* and Δ*C* are the tumor volume changes in treatment and control groups, respectively.

### 2.16 Statistical Analysis

The data are shown as the mean ± standard error (SE). All experiments were conducted in triplicate. One-way and two-way ANOVA (*p < 0.05; **p < 0.001) using Dunnett’s or Tukey’s post hoc analysis were used to calculate the significance between tested groups.

## 3 Results and Discussion

### 3.1 Physicochemical Characterization

The usAuNPs were synthesized from HAuCl_4_·3H_2_O using NaBH_4_ as the reducing agent and alginate as the stabilizing agent (Figure 1a), following the method described in our previous work.^27^ The UV–vis absorption spectrum exhibited a distinct localized surface plasmon resonance (LSPR) band at 511 nm, characteristic of usAuNPs (Figure 1b). TEM revealed that usAuNPs have a uniform size distribution with an average particle diameter of 3.0 nm (Figure 1c–e). DLS measurements further confirmed an average hydrodynamic diameter of 4.8 nm and a ζ potential of −36.8 mV (Table 1).

**Figure 1:**
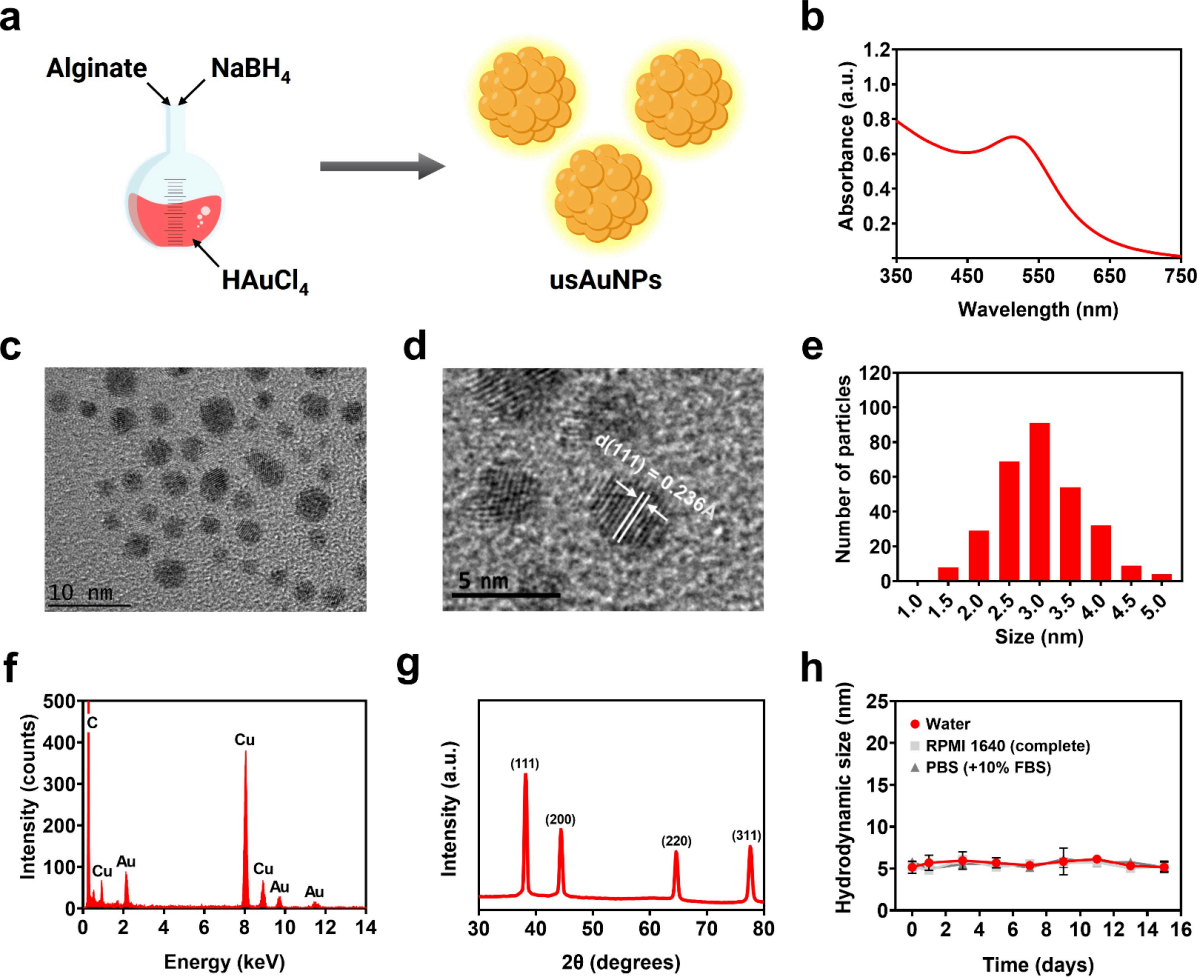
Physicochemical characterization of usAuNPs. (a) Overall scheme of the preparation method of usAuNPs. (b) UV–vis absorption spectrum. (c and d) TEM images at 400,000× and 630,000× magnifications. (e) Core size distribution (*n* = 300). (f) EDX spectrum. (g) XRD pattern. (h) Stability of usAuNPs in various aqueous media.

**Table 1:** Physicochemical Characteristics of usAuNPs^a^

sample	LSPR peak (nm)	core size^b^ (nm)	hydrodynamic size^c^ (nm)	ζ potential (mV)
usAuNPs	511	3.0 (±0.8)	4.8 (±0.9)	–36.8 (±1.9)

a Data are presented as mean ± SE (*n* = 3).

b Obtained from TEM images using the *ImageJ* software (*n* = 300).

c Obtained from DLS.

The elemental composition of the synthesized usAuNPs was verified by EDX spectroscopy. As expected, the EDX spectrum showed a prominent peak at 2.2 eV corresponding to Au (Figure 1f). Additional EDX peaks of carbon (C) and copper (Cu) arose due to the background signals from the carbon-coated copper grids used for sample preparation in TEM analysis. High-resolution TEM measurements indicated a crystal lattice *d* spacing of approximately 2.36 Å, corresponding to the (111) plane of metallic Au. XRD analysis provided further confirmation, showing characteristic Bragg reflections at 38.18°, 44.40°, 64.58°, and 77.57°, which correspond to the (111), (200), (220), and (311) planes of a face-centered-cubic Au lattice structure, respectively^39^ (Figure 1g).

Additionally, the synthesized usAuNPs exhibited excellent aqueous stability in various biologically relevant media (ultrapure water, complete RPMI 1640, and PBS supplemented with 10% FBS). No significant changes in the hydrodynamic size were observed after incubation for up to 14 days at 37 °C (Figure 1h). After 14 days, FTIR analysis was performed on the samples to validate the stability of the alginate coating. The usAuNPs stored in water for 14 days showed characteristic absorption bands at 3278, 1595, 1408, and 1028 cm^–1^, which correspond to O–H, asymmetric COO–, symmetric COO–, and C–O stretching vibrations of alginate at intensities similar to those of freshly synthesized usAuNPs (Figure S1). In contrast, usAuNPs incubated in PBS (with 10% FBS) displayed new absorption peaks at 1635 and 1540 cm^–1^, in addition to the alginate bands, which were attributed to amide I and amide II vibrations of serum proteins adsorbed onto the nanoparticle surface, indicating the formation of a protein corona.^40^ Overall, the findings reflect the stability of usAuNPs under physiological conditions, underscoring their robustness and suitability for biological applications.

### 3.2 Catalytic Activity of usAuNPs: H_2_O_2_ and ^•^OH Generation

The POD-like catalytic activity of usAuNPs was evaluated based on their ability to convert H_2_O_2_ into ^•^OH, using ammonium molybdate colorimetric assay. As shown in Figure 2a, usAuNPs efficiently catalyzed the decomposition of H_2_O_2_ by 67%, 77%, and 93% at concentrations of 50, 100, and 200 μg/mL, respectively, after 24 h of incubation at 37 °C. Moreover, MB was utilized as a sensitive probe to detect the formation of ^•^OH radicals from the decomposition of H_2_O_2_. A gradual decrease in MB absorbance was observed in the presence of 200 μg/mL of usAuNPs and 8 mM H_2_O_2_ (Figure 2b), confirming the effective generation of ^•^OH as a result of POD-like activity. The first-order rate constant for ^•^OH generation was calculated to be 5.7 × 10^–3^ min^–1^ (Figure 2c). Furthermore, the catalytic performance depended on the initial concentration of H_2_O_2_, with higher rates of ^•^OH generation observed as the H_2_O_2_ concentration increased (Figure 2d).

**Figure 2:**
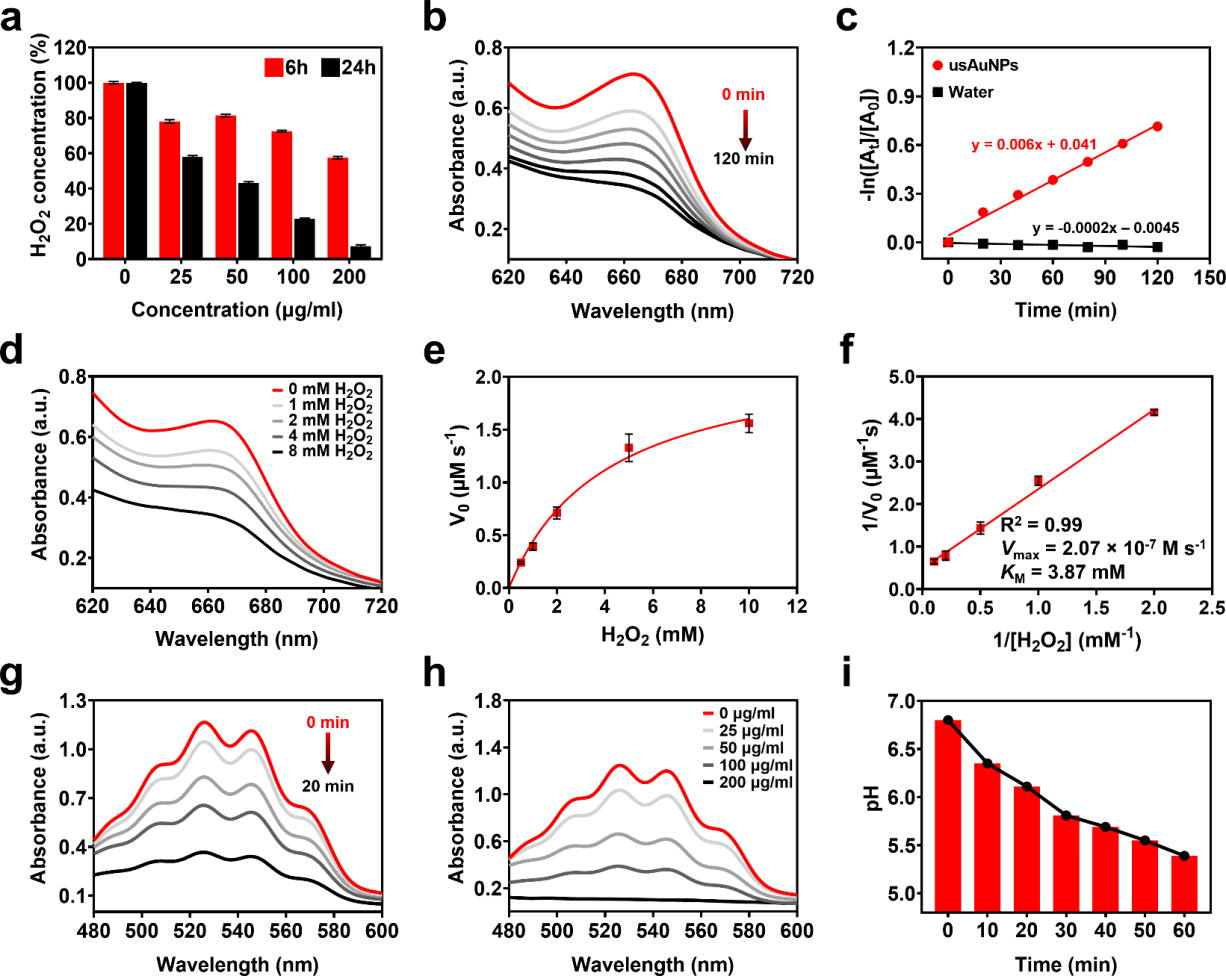
(a) H_2_O_2_ depletion by usAuNPs (0–200 μg/mL) via POD-like activity. Data are represented as the mean ± SE (*n* = 3) normalized to that of the control group. (b) Time-dependent and (d) substrate-dependent ^•^OH generation carried out by usAuNPs (100 μg/mL) after 120 min. (c) First-order reaction rate constant for ^•^OH generation. (e) Michaelis–Menten kinetic curve of TMB oxidation by usAuNPs with varying H_2_O_2_ concentration (0.5–10.0 mM) and (f) corresponding Lineweaver–Burk double-reciprocal plot. Data are represented as mean ± SE (*n* = 3). (g) Time-dependent H_2_O_2_ generation by usAuNPs (100 μg/mL) via GOx-like activity. (h) Effect of the usAuNPs concentration (0–200 μg/mL) on the reduction in KMnO_4_ absorbance in the presence of glucose (500 μg/mL). (i) pH as a function of time for usAuNPs (200 μg/mL) and glucose (500 μg/mL) mixtures.

Kinetic parameters, such as the maximum reaction velocity (*V*_max_) and Michaelis–Menten constant (*K*_M_), were evaluated using TMB assay to provide quantitative insights into the POD-like catalytic activity of usAuNPs. The initial reaction rates of TMB oxidation at various concentrations of H_2_O_2_ (0.5–10 mM) fitted well into the Michaelis–Menten enzyme kinetic model and Lineweaver–Burk double-reciprocal plot, indicating POD-like behavior of usAuNPs (Figure 2e,f). Thus, kinetic parameters *V*_max_ and *K*_M_ were calculated from the Lineweaver–Burk plot. *K*_M_ is defined as a substrate concentration needed to achieve half of *V*_max_ and reflects affinity of the enzyme toward the substrate, with smaller *K*_M_ indicating greater affinity toward the substrate. For usAuNPs, *V*_max_ and *K*_M_ were calculated to be 2.07 × 10^–7^ M s^–1^ and 3.87 mM, reflecting both their strong substrate affinity and high catalytic activity. Compared to the natural enzyme HRP, usAuNPs displayed similar substrate affinity and more than a 2.5-fold greater *V*_max_, suggesting excellent potential as enzyme mimics. Notably, usAuNPs consistently demonstrated comparable or higher affinity and catalytic activity relative to numerous reported Au-containing as well as non-Au nanozymes (Cu-, Pd-, or Pt-based systems; Table S1). These results established usAuNPs as a highly effective POD mimic, exhibiting robust enzyme-like activity and outperforming other reported nanozymes.

Nevertheless, the therapeutic efficacy of nanocatalysts is often limited by insufficient endogenous H_2_O_2_ at the tumor site.^6^ To overcome this limitation, the GOx-like catalytic activity of usAuNPs was evaluated by examining their ability to convert molecular glucose into H_2_O_2_. KMnO_4_ was employed as a probe to monitor the breakdown of glucose and consequent H_2_O_2_ production mediated by usAuNPs. The optical intensity of KMnO_4_ decreased by approximately 90% within 20 min of incubation with usAuNPs, indicating rapid and efficient H_2_O_2_ production (Figure 2g). Moreover, the rate of H_2_O_2_ generation correlated positively with both the incubation time (Figure 2g) and nanoparticle concentration (Figure 2h). In contrast, no reduction in the optical intensity of KMnO_4_ was observed in the absence of usAuNPs (Figure S2). The decrease in the solution pH following the incubation of glucose with usAuNPs further confirmed the production of gluconic acid as a byproduct, validating the GOx-like catalytic mechanism (Figure 2i). Taken together, these findings demonstrate that usAuNPs exhibit dual enzyme-mimicking activities, effectively generating H_2_O_2_ via glucose oxidation (GOx-like) while rapidly decomposing H_2_O_2_ into cytotoxic ^•^OH radicals (POD-like). This unique catalytic dual functionality could potentially induce glucose starvation and simultaneously enhance the catalytic cancer therapy efficacy.

### 3.3 Ultrasound-Triggered ROS Generation

Ultrasound-triggered generation of ROS by usAuNPs was evaluated by using DPBF as a fluorescent probe. DPBF is converted into a nonfluorescent endoperoxide upon reaction with ROS (^1^O_2_ and ^•^OH), allowing quantitative measurement of ROS production.^41^ The sonosensitizing efficiency of usAuNPs (50 μg/mL) was found to be dependent on the ultrasound intensity, with significant generation of ROS observed even at a low ultrasound intensity of 0.1 W/cm^2^ (Figure S3). The production of ROS increased proportionally with the ultrasound intensity, reaching a maximum at 2.0 W/cm^2^. At this ultrasound intensity, usAuNPs induced a 2.4-fold reduction in the optical intensity of DPBF within 5 min of ultrasound irradiation (Figure 3a). In contrast, negligible ROS generation was detected in control samples without nanoparticles under identical experimental conditions (Figure 3b). The rate constant for ROS generation by usAuNPs at an ultrasound intensity of 2.0 W/cm^2^ was determined to be 1.78 × 10^–1^ min^–1^. To elucidate the specific ROS generated by usAuNPs, the nanoparticles were irradiated in the presence of mannitol (^•^OH scavenger) and histidine (scavenger of both ^•^OH and ^1^O_2_). As shown in Figure 3b, mannitol caused only a partial reduction in DPBF depletion, whereas histidine completely suppressed ROS generation, indicating that usAuNPs predominantly generate ^1^O_2_ under ultrasound irradiation.

**Figure 3:**
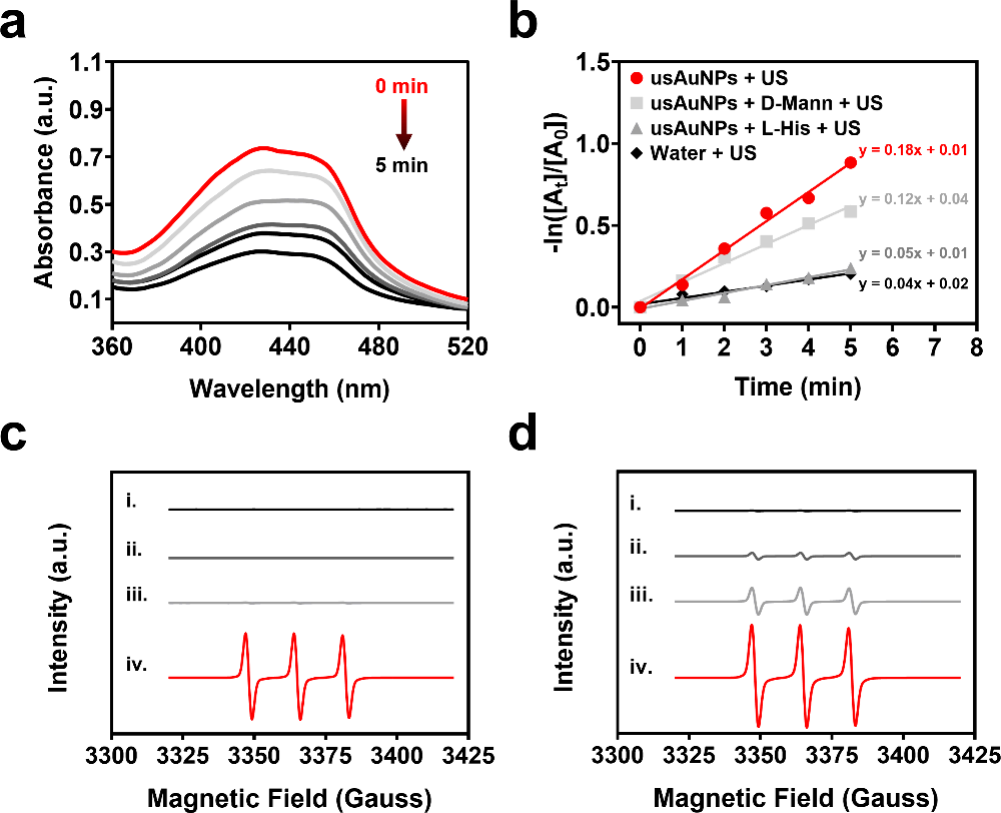
Ultrasound-triggered ROS production by usAuNPs. (a) Time-dependent DPBF absorbance depletion by usAuNPs (50 μg/mL) under ultrasound irradiation (2.0 W/cm^2^). (b) First-order rate constant under different treatments: 50 μg/mL usAuNPs, 50 μg/mL usAuNPs + 25 mM mannitol (d-Mann), 50 μg/mL usAuNPs + 25 mM histidine (l-His) and water under ultrasound irradiation (2.0 W/cm^2^). (c) ^1^O_2_ generation by (i) water, (ii) water + ultrasound, (iii) usAuNPs, and (iv) usAuNPs + ultrasound detected using ESR spectra. (d) Effect of usAuNPs at (i) 10 μg/mL, (ii) 50 μg/mL, (iii) 100 μg/mL, and (iv) 200 μg/mL on ^1^O_2_ generation under ultrasound (2.0 W/cm^2^, 3 min).

ESR spectroscopy was employed to further confirm the production of ^1^O_2_. The characteristic ESR peak ratio of 1:1:1, indicative of ^1^O_2_ generation, was clearly observed upon ultrasound irradiation of usAuNPs (Figure 3c). The magnitude of ^1^O_2_ generation strongly correlated to the nanoparticle concentration. The highest ESR signal was obtained with 200 μg/mL usAuNPs, which showed ESR peak intensities approximately 16-fold and 4-fold greater than those observed at concentrations of 50 and 100 μg/mL, respectively (Figure 3d). Notably, control experiments conducted with water alone and with water under ultrasound irradiation showed no detectable ^1^O_2_ production. Similarly, negligible ^1^O_2_ production was observed for 200 μg/mL usAuNPs without ultrasound exposure. These findings are consistent with previous studies,^27,28,42^ further supporting the excellent sonosensitizing properties of usAuNPs and highlighting their potential as highly effective candidates for SDT.

### 3.4 Biocompatibility Evaluation of usAuNPs

The biocompatibility of usAuNPs was systematically evaluated through cytocompatibility and hemocompatibility assays to establish their biosafety profile. Cytotoxicity was assessed using murine fibroblast cells (L929) via MTT assay. Cells were exposed to various concentrations of usAuNPs (0–200 μg/mL) for 24 h, and the cell viability was subsequently measured. As shown in Figure 4a, treatment with usAuNPs did not significantly affect the cell viability (*p* > 0.05), even at the highest concentration tested (200 μg/mL), demonstrating excellent cytocompatibility.

**Figure 4:**
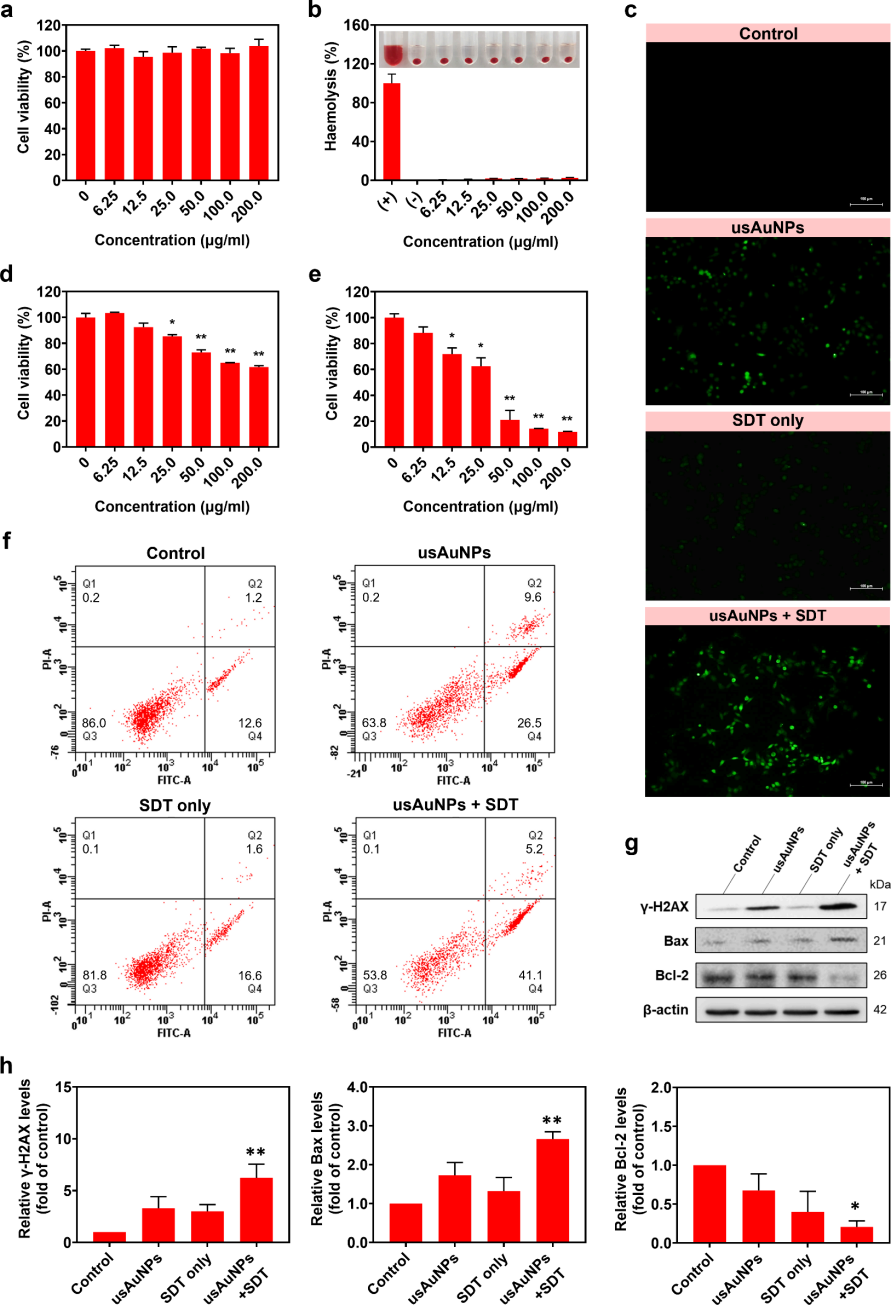
(a) Cytocompatibility of usAuNPs on L929 murine fibroblasts after 24 h. (b) Hemocompatibility of usAuNPs after 5 h of incubation with murine RBCs. (c) ROS staining of 4T1 breast cancer cells after various treatments using DCFH-DA. Cell viability of 4T1 after 24 h of (d) catalytic therapy and (e) SDT/catalytic therapy by usAuNPs. Data are represented as mean ± SE (*n* = 3) normalized to that of the control group (*p < 0.05 and **p < 0.001 obtained via one-way ANOVA using Dunnett’s posthoc test). (f) Cell death pattern of 4T1 in different treatment groups after 10 h. (g and h) Western blot analysis of γ-H2AX, Bax, and Bcl-2 expressions in 4T1 cells after different treatments. The data were calculated as the ratio of the target protein expression versus β-actin, normalized to that of the control group and represented as mean ± SE (*n* = 3; *p < 0.05 and **p < 0.001 obtained using an unpaired Student’s *t* test).

Hemocompatibility was further evaluated by incubating murine RBCs with varying concentrations of usAuNPs (0–200 μg/mL) for 5 h. Hemolysis levels were determined by measuring the release of hemoglobin. As illustrated in Figure 4b, minimal hemolysis was observed with usAuNPs, even at the highest concentration (200 μg/mL), with less than 3% of RBCs lysis recorded. According to the ASTM 2524-22 standard, hemolysis levels exceeding 5% are considered harmful to erythrocytes.^43^ Therefore, these results indicate that usAuNPs exhibit negligible hemolytic activity and are suitable candidates for safe systemic administration.

### 3.5 Intracellular ROS Generation

Intracellular oxidative stress by usAuNPs was assessed using DCFH-DA assay. DCFH-DA is a cell-permeable nonfluorescent probe that becomes oxidized by intracellular ROS into a highly fluorescent derivative, 2′,7′-dichlorofluorescein (DCF). As shown in Figure 4c, weak fluorescence signals were observed in the control and ultrasound-only groups, indicating minimal ROS production. In contrast, cells treated with usAuNPs alone exhibited a noticeable increase in green fluorescence, attributed to the intracellular catalytic generation of ^•^OH radicals by the nanoparticles. As anticipated, the highest fluorescence intensity was observed in cells treated with usAuNPs combined with ultrasound irradiation (2.0 W/cm^2^, 2 min), indicating a significant enhancement in intracellular ROS production. Collectively, these results confirm the excellent sonosensitizing and catalytic properties of usAuNPs in promoting intracellular oxidative stress.

### 3.6 *In Vitro* Cytotoxicity and SDT

The cytotoxicity of usAuNPs was evaluated by using murine triple-negative breast cancer (4T1) cells through MTT assay. Cells were treated with various concentrations of usAuNPs (0–200 μg/mL) for 24 h. As shown in Figure 4d, no significant cytotoxicity was observed in L929 fibroblasts treated with usAuNPs, even at the highest tested concentration (200 μg/mL), confirming their excellent biocompatibility. However, 4T1 breast cancer cells exhibited moderate cytotoxicity under identical treatment conditions, with approximately 38% cell death at 200 μg/mL (Figure 4d). This observation can be attributed to the catalytic activity of usAuNPs in the mildly acidic tumor microenvironment. Remarkably, when combined with SDT, usAuNPs showed significant dose-dependent cytotoxicity (*p* < 0.001), inducing 79%, 86%, and 88% cell death at concentrations of 50, 100, and 200 μg/mL, respectively (Figure 4e).

Flow cytometry analysis was performed to characterize the type of cell death induced by the combined SDT/catalytic therapy. At 6 h post-treatment, neither SDT alone nor usAuNPs alone led to a significant increase in apparent cell death relative to the control group (viabilities of 98% and 97%, respectively; Figure S4). However, the cells exposed to usAuNPs alone exhibited more pronounced cytotoxicity at 10 h of incubation, with viability declining to 64% and the apoptotic population rising sharply from 2.8% to 36%, indicating that the cytotoxic effect of usAuNPs manifests in a delayed yet progressive manner, likely attributed to gradual cellular internalization and cumulative oxidative stress (Figure 4f). In contrast, combined SDT/catalytic therapy exhibited an immediate anticancer effect, causing a significant reduction in viability (11%) after 6 h post-treatment, accompanied by apparent increases in early (81.2%) and late (8.1%) apoptotic cell populations, with minimal necrotic cell death detected (Figure S4). These findings suggest that combined SDT/catalytic therapy using usAuNPs predominantly induces apoptotic death in breast cancer cells, underscoring its potential for targeted and controlled tumor eradication.

To further elucidate the apoptosis mechanism underlying the combined therapy, key apoptotic markers including phosphorylated histone H2AX (γ-H2AX), Bcl-2, and Bax were examined by Western blot analysis (Figure S5). The level of γ-H2AX, a sensitive marker of DNA double-strand breaks, increased by approximately 6.2-fold following the combined treatment, indicating significant DNA damage (Figure 4g,h). Additionally, combined therapy markedly elevated the expression of proapoptotic Bax (2.7-fold increase) and simultaneously reduced the level of antiapoptotic Bcl-2 (4.8-fold decrease) compared to untreated controls. The Bax/Bcl-2 ratio is a crucial regulator of mitochondrial apoptosis, promoting cytochrome *c* release and subsequent activation of caspase cascades, leading to apoptosis.^44^ Collectively, these findings demonstrate that combined SDT and catalytic therapy mediated by usAuNPs triggers ROS-induced DNA damage and induces apoptosis through the mitochondrial pathway. Overall, these results highlight the strong therapeutic potential of usAuNPs as sonocatalytic agents in enhancing the cancer treatment efficacy.

### 3.7 *In Vivo* Acute Toxicity and Biodistribution

Prior to evaluation of the *in vivo* therapeutic efficacy of combined SDT/catalytic therapy, the acute toxicity of usAuNPs was assessed in female BALB/c mice. The mice received a single intravenous administration of usAuNPs at doses ranging from 0 to 16 mg/kg and were closely monitored for signs of toxicity over a 14-day period (Figure 5a). No significant changes in body weight were observed in any treatment group throughout the study period (Figure 5b). Furthermore, histopathological examination of major organs revealed no obvious pathological abnormalities or toxicity associated with the treatment, even at the highest dose (16 mg/kg), confirming the excellent biosafety of usAuNPs (Figure 5c).

**Figure 5:**
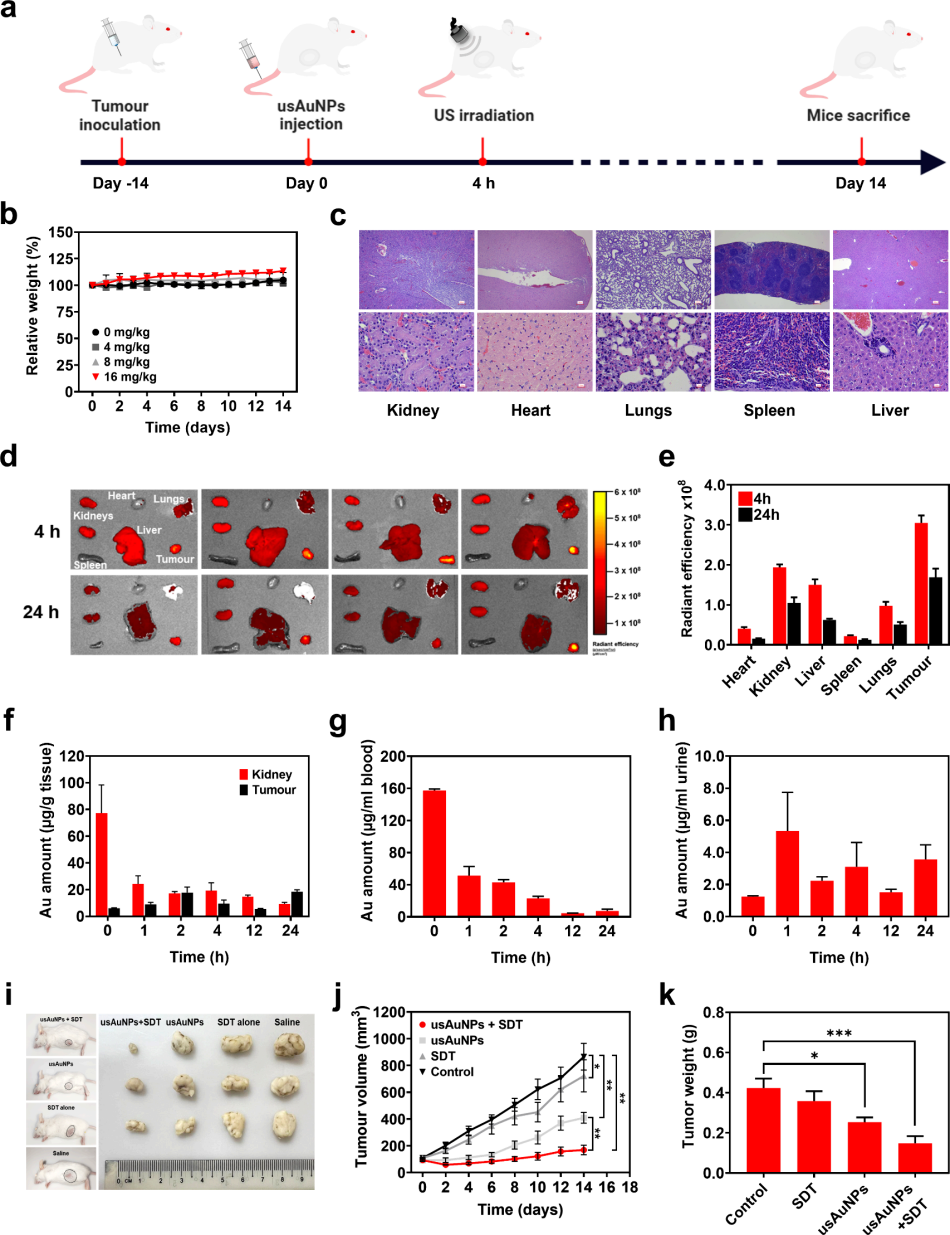
(a) Schematic illustration of the operation procedure of SDT/catalytic therapy on 4T1 tumor-bearing female BALB/c mice (6–8 weeks old). (b and c) *In vivo* acute toxicity from the single intravenous dose of 16 mg/kg usAuNPs (*n* = 3). (d and e) Average fluorescent signal of heart, kidney, liver, spleen, lungs, and tumor 4 and 24 h post-treatment measured by an IVIS spectrum system (*n* = 6). Gold concentration in (f) kidney and tumor, (g) blood, and (h) urine at 0, 1, 2, 4, 12, and 24 h after intravenous injection of 16 mg/kg usAuNPs measured by ICP-MS. Data are represented as mean ± SE, normalized to a weight of tissue or a volume of liquid sample. (i) Representative photographs of tumor-bearing mice and tumors after various treatments. (j) Tumor growth curves and (k) weight of tumors after various treatments (*n* = 6). Data are represented as mean ± SE (*p < 0.05 and **p < 0.001 obtained by two-way ANOVA using Tukey’s post hoc analysis).

Tissue biodistribution of usAuNPs was first assessed by IVIS imaging. At 4 and 24 h postinjection, the highest fluorescence signals were consistently detected in tumor tissues, followed by kidneys, liver, and lungs. This preferential tumor accumulation suggests tumor-targeting capability of usAuNPs, likely facilitated by the enhanced permeability and retention (EPR) effect (Figure 5f,g). Moreover, fluorescence signals in all tested organs decreased on average by approximately 2.2-fold within 24 h, suggesting rapid whole-body clearance of usAuNPs, thus minimizing potential long-term organ toxicity.

The pharmacokinetic profile of usAuNPs was further assessed by quantifying their distribution in the kidney, tumor, blood, and urine using ICP-MS. The results revealed a high initial renal burden (77.4 μg/g of tissue) that progressively declined over 24 h, indicating a fast clearance of nanoparticles (Figure 5f). In contrast, usAuNPs exhibited a rapid accumulation in the tumor, reaching a peak concentration of 17 μg/g within 2 h after injection. At 24 h, accumulation of usAuNPs in tumor tissues exceeded that in the kidneys, corroborating the tumor-uptake dynamics observed by fluorescence imaging (Figure 5f). Discrepancies between IVIS and ICP-MS measurements were attributed to fluorescence attenuation often reported in strongly absorbing organs (e.g., liver and kidney), resulting in the underestimation of fluorescence signals relative to more superficial tissues such as tumor.^45,46^ Thus, ICP-MS provided absolute quantitative measurements of nanoparticle biodistribution, whereas fluorescence imaging offered complementary insights into spatial localization and temporal kinetics. In addition, usAuNPs displayed a fast blood elimination rate, with concentration decreasing from 157.3 to 7.2 μg/mL within 24 h after administration, and were detectable in urine samples up to 24 h (Figure 5g,h). These observations align closely with previously published studies on the *in vivo* biokinetics of usAuNPs.^47^

### 3.8 *In Vivo* Combined SDT/Catalytic Therapy

The therapeutic efficacy of combined SDT/catalytic therapy mediated by usAuNPs was evaluated *in vivo* using a murine tumor model. Tumor volumes were monitored over a 2-week treatment period (Figure 5h). In both the control (saline) and SDT-alone groups, tumors rapidly grew, exhibiting approximately 8-fold and 6.6-fold increases in volume, respectively. SDT alone showed a minimal TGI of only 18.8% compared to the saline group (Figure 5h,i), which might be attributed to damage from mild hyperthermia generated during ultrasound irradiation (Figures S6–S8). Treatment with usAuNPs alone (16 mg/kg) resulted in significant initial suppression of tumor growth for up to 6 days postinjection (TGI = 85.7%); however, tumor volumes gradually increased thereafter, reaching approximately 4-fold by day 14, corresponding to a TGI of 59.0% compared to the saline control.

Notably, combined SDT/catalytic therapy significantly inhibited tumor growth throughout the entire treatment period, achieving approximately 90% TGI by day 14 (Figure 5h). Tumor weight measurements at the end point were consistent with the observed tumor volume reduction after different treatments, showing significant reduction (*p* < 0.05) in tumor weights detected in mice treated with usAuNPs only and usAuNPs + SDT. Combined SDT/catalytic therapy resulted in nearly a 2.9-fold reduction in the tumor weight compared to the control group. Notably, no acute indicators of toxicity, including weight loss, behavioral alterations, or significant temperature elevation relative to the SDT only group, were detected, highlighting the excellent biosafety profile of usAuNP-mediated combined SDT/catalytic therapy (Figures S6–S9). Collectively, these findings provide compelling *in vivo* evidence that usAuNPs serve as highly effective agents for combinatorial SDT and catalytic therapy against solid tumors.

The combination of catalytic therapy and SDT has shown promising therapeutic effects against solid tumors, prompting the further development of nanocatalysts with synergistic enzyme-mimicking activities.^10,20^ Gold-based nanomaterials have previously been demonstrated to possess GOx-like^48,49^ and POD-mimicking activities.^23,50^ However, larger gold nanoparticles often exhibit prolonged retention in organs such as the liver, potentially leading to long-term toxicity.^51^ In contrast, ultrasmall nanoparticles (<5 nm in diameter) exhibit rapid renal clearance, reducing risks of organ accumulation and related toxicity.^52^ Furthermore, our previous work demonstrated that usAuNPs generate substantial amounts of ROS under ultrasound stimulation.^27^ Consequently, usAuNPs were selected in this study as multifunctional nanozymes and nanosonosensitizers, integrating intrinsic enzyme-mimicking and sonosensitizing properties into a single “three-in-one” nanocatalyst platform.

Experimentally, usAuNPs exhibited efficient catalytic decomposition of H_2_O_2_ into highly cytotoxic ^•^OH radicals (*K*_M_ = 3.89), comparable in performance to previously reported nanocatalytic systems.^26,34,35,53^ However, the catalytic performance is largely dependent on the concentration of available H_2_O_2_. Given the limited endogenous levels of H_2_O_2_ in tumors, sustained catalytic output remains to be a challenge.^6^ To address this limitation, usAuNPs were further demonstrated to catalyze glucose oxidation, generating H_2_O_2_ continuously. This glucose-dependent H_2_O_2_ generation is supported by literature highlighting glucose as an abundant substrate in tumors due to their increased glycolytic metabolism (Warburg effect), making glucose ideal for sustained catalytic reactions and potentially inducing glucose depletion in tumors.^54–56^ Indeed, our data showed significant glucose depletion with concomitant increases in H_2_O_2_ production and gluconic acid formation, evidenced by decreased medium pH. While these results strongly indicate GOx-mimicking catalytic activity, further mechanistic studies investigating downstream metabolic effects such as ATP depletion *in vivo* remain to be explored.^57^

In addition to their catalytic functions, the sonosensitizing capability of usAuNPs represents another critical feature. Previous reports have highlighted the sonosensitizing properties of usAuNPs as a component of complex nanostructures, serving as electron sinks to prevent electron–hole recombination and enhance the sonocatalytic performance.^58,59^ However, studies investigating the sonosensitizing performance of usAuNPs alone are rare. Our results clearly demonstrated that standalone usAuNPs possess substantial sonosensitizing capabilities. Under 1 MHz ultrasound irradiation, usAuNPs generated a significant amount of ^1^O_2_ with a rate constant of 1.78 × 10^–1^ min^–1^. This efficiency surpasses previously reported Au-based nanocomplexes such as Au–Ir­(III) nanoparticles,^28^ Au-TiO_2_ nanocomposites,^42^ Au-ZnO-bridged carbon nanosheets,^59^ Au-loaded ZnO nanorods,^31^ and Au-Cu_2_S nanohybrids.^32^ Additionally, the observed superior sonodynamic performance aligns with the concept of the nanoparticle size influencing the ROS generation efficiency, where smaller AuNPs typically exhibit enhanced catalytic and sonodynamic efficiencies.^42^ At dimensions below 2–3 nm, AuNPs exhibit pronounced quantum confinement effects, resulting in discrete molecular-like energy levels and a HOMO–LUMO gap.^60,61^ This altered electronic structure may promote more effective electron–hole separation under ultrasound excitation, thereby facilitating intersystem crossing and ^1^O_2_ production.^62,63^ Future studies incorporating theoretical approaches such as density functional theory calculations will be valuable to further clarify the relationship between the gold nanoparticle size, quantum confinement, and sonodynamic performance.

Combined SDT/catalytic therapy significantly enhanced cellular DNA damage, as evidenced by the increased expression of phosphorylated histone H2AX (γ-H2AX), a reliable marker for DNA double-strand breaks. Literature indicates that significant DNA damage often triggers downstream apoptotic signaling pathways, including modulation of proapoptotic Bax and antiapoptotic Bcl-2 proteins, subsequently activating mitochondrial apoptosis pathways.^44,64^ Consistent with these findings, the combined treatment markedly elevated Bax expression while reducing Bcl-2 expression, confirming apoptosis predominantly through mitochondrial mechanisms.

*In vivo* evaluation further supported the potential clinical applicability of usAuNPs. The nanoparticles demonstrated excellent tolerance in BALB/c mice at doses up to 16 mg/kg, showing minimal acute organ toxicity, likely due to their rapid renal clearance confirmed by fluorescence imaging. Literature similarly emphasizes that nanoparticles smaller than 5 nm effectively extravasate and rapidly clear from the system, enhancing their tumor penetration and limiting long-term toxicity.^65^ Biodistribution studies showed an apparent preferential tumor accumulation of usAuNPs, with peak tumor concentrations achieved within hours postinjection, as well as rapid blood clearance. However, the long-term pharmacokinetics of these ultrasmall nanoparticles was not properly evaluated in this study, necessitating future investigations to be made before establishing these usAuNPs as clinically safe nanomaterials.

Although a single 16 mg/kg dose of usAuNP alone initially suppressed tumor growth, the therapeutic effect diminished over time, possibly due to clearance mechanisms previously reported for ultrasmall nanoparticles.^47^ Therefore, strategies involving repeated or timely dosing regimens could be beneficial, although such protocols require systematic investigation for optimized efficacy and safety profiles. Nevertheless, the current single-dose combined SDT/catalytic therapy achieved superior tumor suppression (approximately 90% inhibition) compared to monotherapies, without causing detectable systemic toxicity.

The translational potential of usAuNPs is reinforced by their compatibility with a therapeutic ultrasound system already widely employed in clinical practice.^66^ Under the applied parameters, only a minimal thermal rise was observed, confirming that tumor ablation was driven primarily by ROS generation rather than hyperthermia. This nonthermal, ROS-specific mechanism minimizes collateral tissue damage, underscoring both the safety and translational relevance of SDT with usAuNPs. Beyond direct ROS-mediated tumor killing, recent studies suggest that combining sonocatalytic nanomaterials or nanozymes with immune modulation can further enhance therapeutic efficacy.^67,68^ Future endeavors integrating usAuNPs with immunoregulatory strategies may, therefore, provide synergistic benefits and broaden their therapeutic relevance.

Collectively, our results provide robust evidence supporting usAuNPs as promising candidates for multifunctional catalytic and sonodynamic therapies, highlighting their potential for clinical translation in cancer treatment strategies. Extended *in vivo* analyses (e.g., immunohistochemistry, TUNEL, survival/recurrence studies, and long-term biodistribution) that would further reinforce the mechanistic conclusions remain important directions for future work, which can build upon the compelling preliminary evidence presented here.

## 4 Conclusions

In summary, we successfully synthesized usAuNPs possessing both catalytic and sonosensitizing properties for effective combined catalytic and sonodynamic cancer therapy. These usAuNPs demonstrated robust POD-like activity, efficiently converting H_2_O_2_ into highly toxic ^•^OH, and exhibited GOx-like activity, continuously generating H_2_O_2_ through glucose oxidation, potentially inducing glucose depletion in cancer cells. Additionally, usAuNPs exhibited exceptional sonosensitizing capability under ultrasound irradiation, achieving a rate constant of ^1^O_2_ generation several-fold higher than previously reported Au-based nanocatalysts. Both *in vitro* and *in vivo* results clearly demonstrated that the combined catalytic therapy and SDT mediated by usAuNPs significantly inhibited breast cancer growth following a single administration combined with ultrasound exposure without causing detectable systemic or organ toxicity. These findings collectively underscore the promise of usAuNPs as safe and highly effective agents for synergistic sonodynamic and catalytic cancer therapies, providing a solid foundation for future translational research, including multidose therapeutic strategies and mechanistic explorations of intracellular responses.

## Supplementary Material



## Notes

FTIR spectra of usAuNPs after 14-day storage in physiological conditions, effect of the ultrasound intensity on ROS production by usAuNPs, cell death pattern analysis after a 6 h treatment of usAuNPs, uncropped Western blot images, intratumoral temperature measurements during ultrasound treatment, changes in body weight of mice in different treatment groups, and table of various enzyme-mimicking nanomaterials (PDF)
