# Supplementary material for: Ultrasmall Gold Nanoparticles as “Three-in-One” Enzyme-Mimicking Nanocatalysts for Combined Sonodynamic/Catalytic Therapy in Breast Cancer
Source: ACS Appl Mater Interfaces. 2025 Dec 4;17(50):67529–42. doi: 10.1021/acsami.5c15866 (PMC12723636; doi:10.1021/acsami.5c15866)
Supplement: Supplementary file 1 [file am5c15866_si_001.pdf]

## Supporting information

# Ultrasmall gold nanoparticles as "three-in-one" enzyme-mimicking nanocatalysts for combined sonodynamic/catalytic therapy in breast cancer

*Adilet Beishenaliev*[a,‡], *Yean Leng Loke*[b,‡], *Chung-Yin Lin*[c,‡], *Sook Jing Goh*[a,d], *Jaya Seema*[d], *Yuhan Huang*[d,e], *Xin Yun Lim*[a], *Yu-Wen Chen*[f], *Bey Fen Leo*[g,h,i,j], *Chia-Yu Chang*[k], *Lip Yong Chung*[l], *Chia-Ching Chang*[k,m,n,o,p], *Yin Yin Teo*[b,*], *Dennis W. Hwang*[d,f,*], *Lik Voon Kiew*[a,i,j,k*]

[a]Department of Pharmacology, Faculty of Medicine, Universiti Malaya, 50603 Kuala Lumpur, Malaysia.

[b]Department of Chemistry, Faculty of Science, Universiti Malaya, 50603 Kuala Lumpur, Malaysia

[c]Research Center for Radiation Medicine, Chang Gung University, 333 Taoyuan, Taiwan.

[d]Institute of Biomedical Sciences, Academia Sinica, 11529 Taipei, Taiwan.

[e]College of Life Sciences, National Yang Ming Chiao Tung University, 30010 Hsinchu, Taiwan.

[f]Biomedical Translation Research Center, Academia Sinica, 11529 Taipei, Taiwan.

[g]Department of Molecular Medicine, Faculty of Medicine, Universiti Malaya, 50603 Kuala Lumpur, Malaysia.

[h]Nanotechnology & Catalysis Research Centre (NANOCAT), Institute for Advanced Studies, Universiti Malaya, 50603 Kuala Lumpur, Malaysia.

[i]Universiti Malaya Research Centre for Biopharmaceuticals and Advanced Therapeutics (UBAT), Faculty of Medicine, Universiti Malaya, 50603 Kuala Lumpur, Malaysia.

[j]Center of Excellence for Innovative Medical Devices, University Malaya, 50603 Kuala Lumpur, Malaysia.

[k]Department of Biological Science and Technology, College of Engineering Bioscience, National Yang Ming Chiao Tung University, 30068 Hsinchu, Taiwan.

[l]Department of Pharmaceutical Chemistry, Faculty of Pharmacy, Universiti Malaya, 50603 Kuala Lumpur, Malaysia.

[m]Center for Intelligent Drug Systems and Smart Bio-devices (IDS$^2$B), National Yang Ming Chiao Tung University, 30068 Hsinchu, Taiwan.

[n]Department of Electrophysics, National Yang Ming Chiao Tung University, 30010 Hsinchu, Taiwan.

[o]Institute of Physics, Academia Sinica, Nankang, 11529 Taipei, Taiwan.

[p]Brain Research Center, National Tsing Hua University, 300044 Hsinchu, Taiwan.

‡: These authors contributed equally to this work.

*Corresponding authors: yinyinteo@um.edu.my (Yin Yin Teo), dwhwang@ibms.sinica.edu.tw (Dennis W. Hwang), lvkiew@um.edu.my (Lik Voon Kiew).

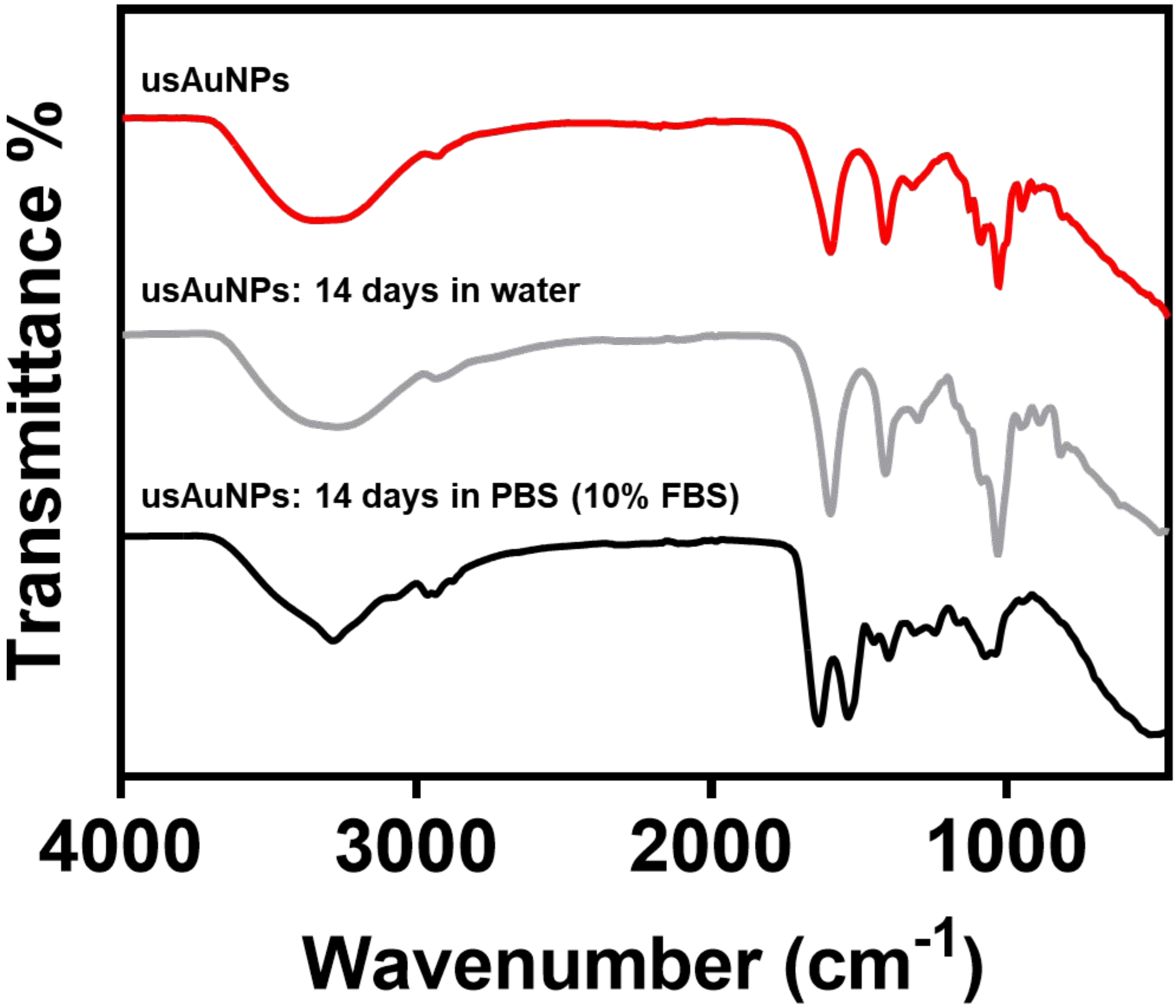


**Figure S1**. FTIR spectra of freshly synthesised usAuNPs and of usAuNPs after 14-day storage at 37 °C in either water or PBS supplemented with 10% FBS.

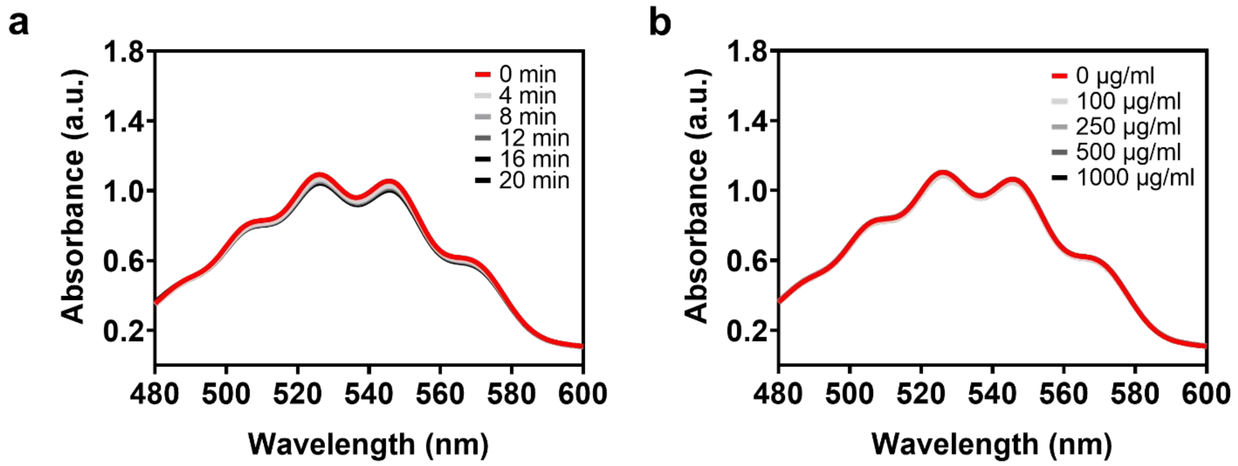


**Figure S2.** (a) Reduction in $KMnO_4$ absorbance mixed with 500 µg/ml glucose. (b) $KMnO_4$ absorbance following the incubation with 0 – 1000 µg/ml glucose for 20 min. No significant reduction in $KMnO_4$ absorbance was detected in absence of usAuNPs.

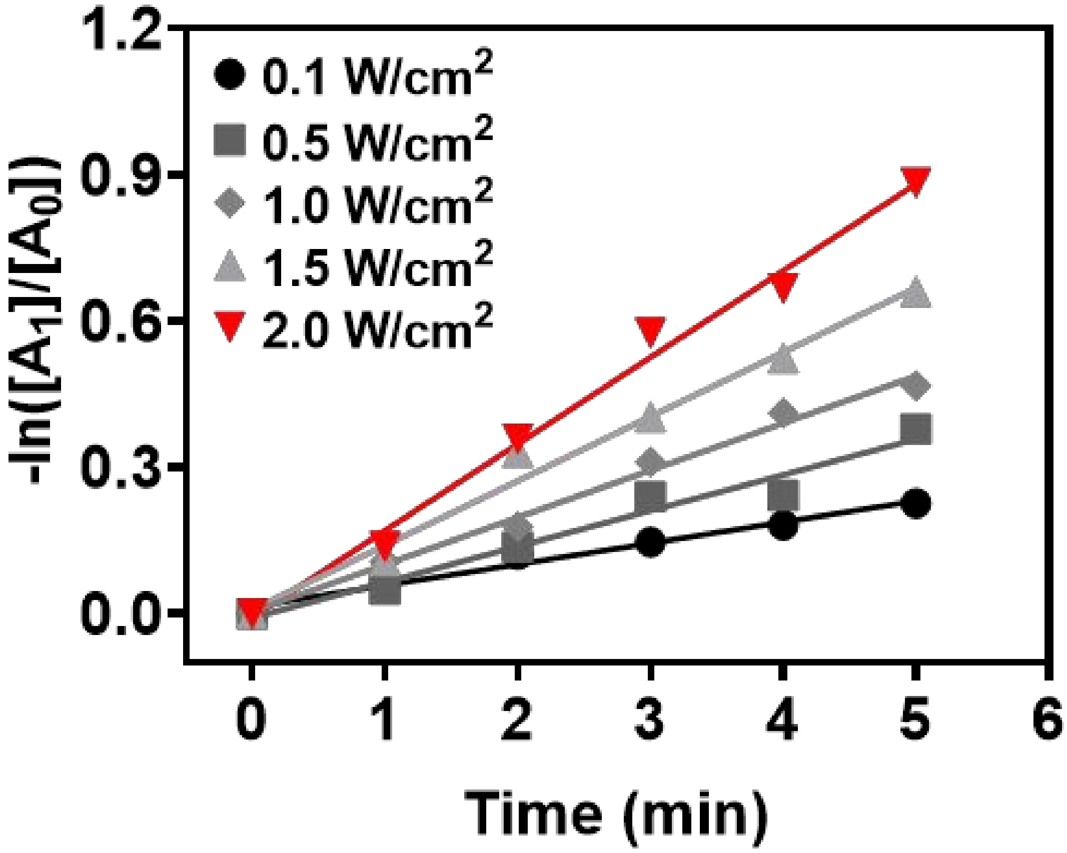


**Figure S3.** Effect of ultrasound intensity (0.1 – 2.0 W/cm$^2$) of ROS production by usAuNPs (50 μg/ml) using DPBF as a probe.

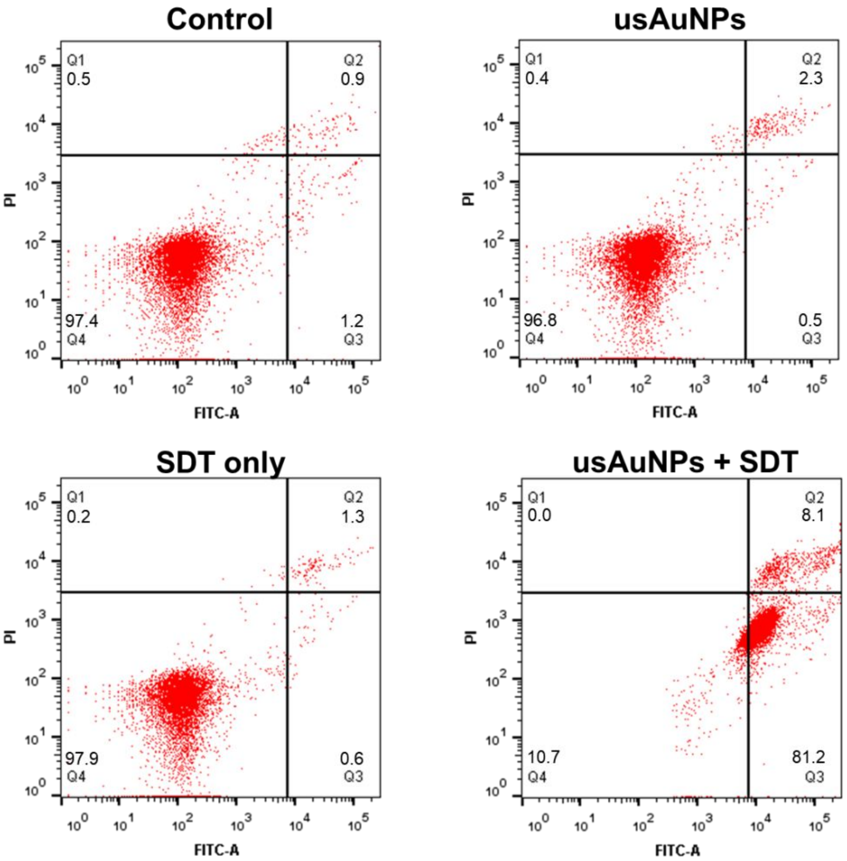


**Figure S4.** Cell death pattern of 4T1 in different treatment groups after 6 h.

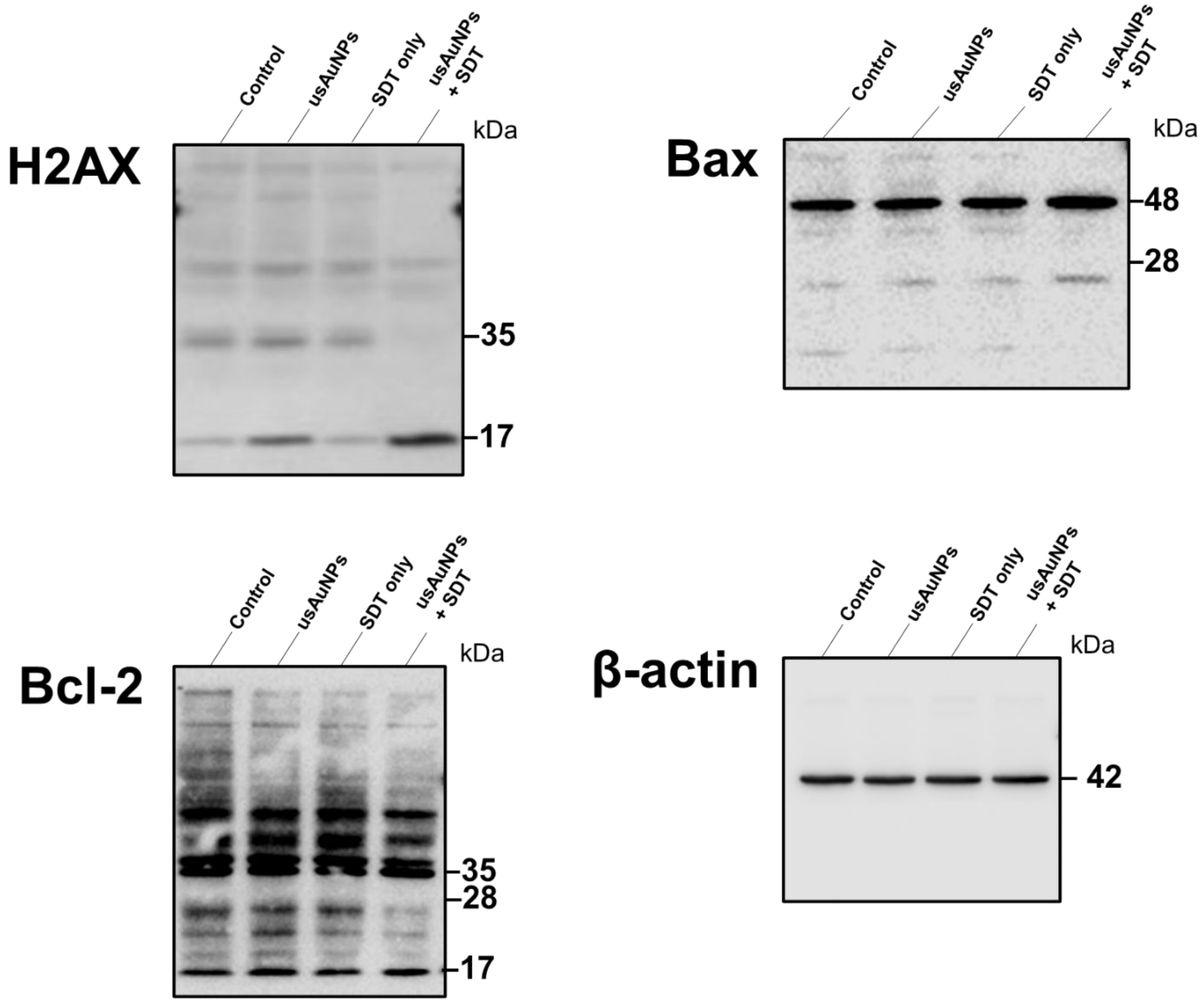


**Figure S5.** Uncropped Western Blot images of Figure 4g. SDT: Sonodynamic therapy

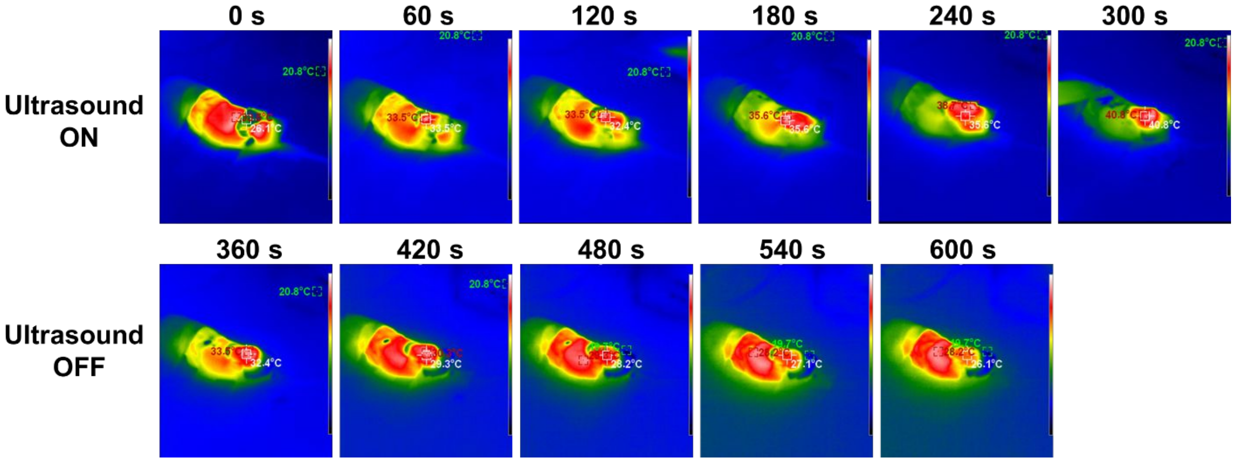


**Figure S6.** Thermal images of 4T1-tumour-bearing BALB/c mice injected with saline and irradiated with ultrasound (2 W/cm$^2$, 100% duty cycle, 5 min).

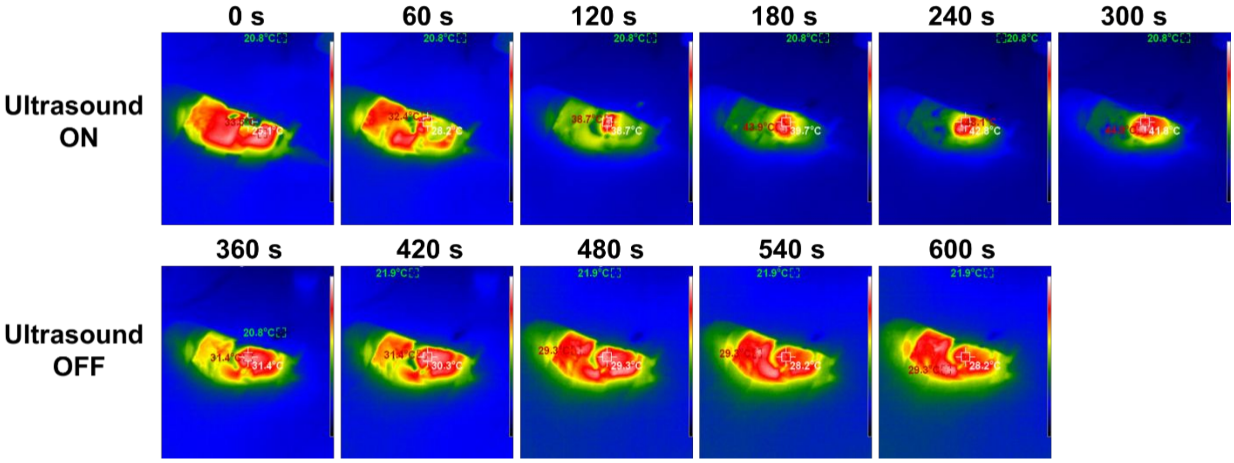


**Figure S7.** Thermal images of 4T1-tumour-bearing BALB/c mice injected with 16 mg/kg usAuNPs and irradiated with ultrasound (2 W/cm$^2$, 100% duty cycle, 5 min).

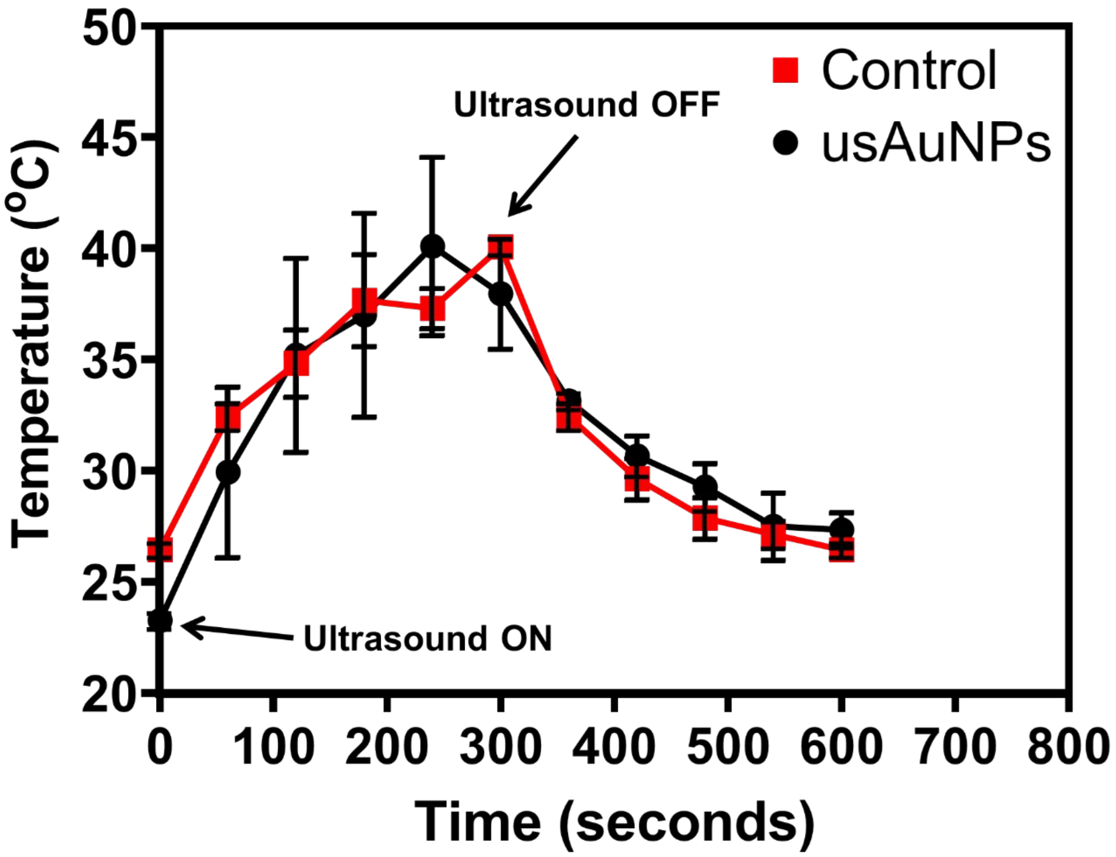


**Figure S8.** Change in tumour temperature during ultrasound treatment (2 W/cm$^2$, 100% duty cycle) in BALB/c mice injected with saline (control) or 16 mg/kg usAuNPs. Ultrasound irradiation was applied for 5 min, after which the transducer was switched off. Tumour temperature was monitored for an additional 5 min, with measurements acquired at 60-second intervals.

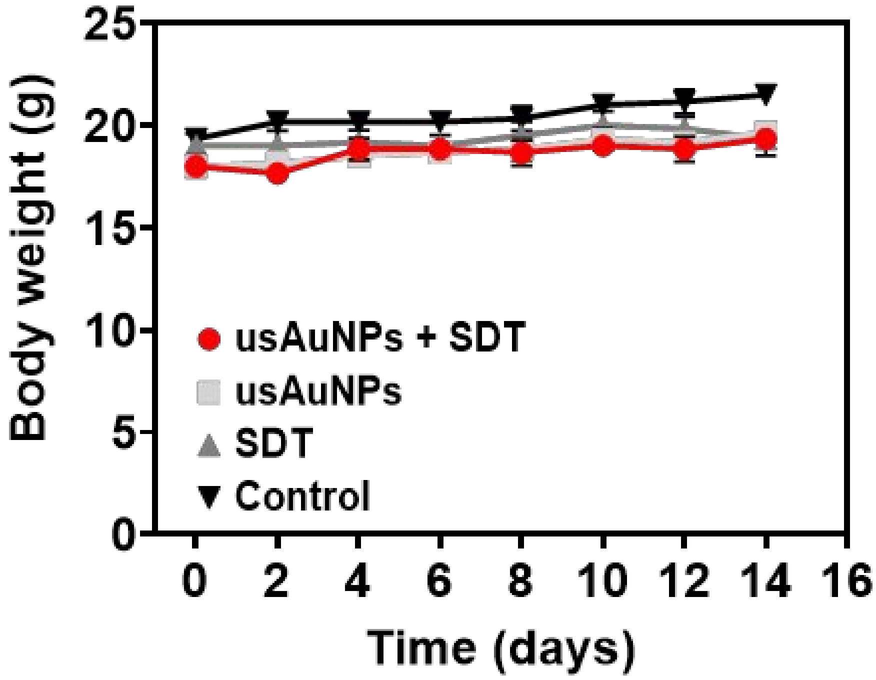


**Figure S9.** Changes in body weights of BALB/c in different treatment groups throughout 14 days of monitoring (n = 6).

**Table S1. Comparison of peroxidase-like performance of various enzyme-mimicking nanomaterials**

| Catalyst | Description | $K_m$ (mM) | $V_{max}$ ($10^{-8}$ M $s^{-1}$) | Reference |
|---|---|---|---|---|
| usAuNPs | Alginate-stabilised ultrasmall gold nanoparticles | 3.89 | 20.7 | This work |
| HRP | Horseradish peroxidase | 3.70 | 8.7 | 1 |
| CuO-Au | Copper oxide-gold nanoalloy | 4.08 | 0.01 | 2 |
| Au@BNSs | Gold nanoparticle-decorated borophene nanosheet | 3.90 | 27.4 | 3 |
| AuMs | Gold nanoparticle-intercalated mesoporous silica | 78.60 | 9.9 | 4 |
| MSN-AuNPs | Mesoporous silica-supported gold nanoparticle | 15.81 | 17.3 | 5 |
| $Fe_3O_4$@$SiO_2$-$NH_2$-Au@$Pd_{0.30}$NPs | Nanomagnet-silica shell decorated with gold@palladium nanoparticle | 3.5 | 6.78 | 6 |
| Au–NP$Fe_2O_3$NC | Gold-loaded nanoporous ferric oxide nanocube | 138.5 | 4.77 | 7 |
| AuNCs-$Pb^{2+}$ | $Pb^{2+}$-induced aggregation of gold nanocluster | 30.8 | 3.39 | 8 |
| $Au_{21}Pd_{79}$ | Palladium nanosheet-supported gold nanoparticle | 5.89 | 8.19 | 9 |
| Au@$Pt_{3.15}$ NPs | Porous gold@platinum nanoparticle | 6.79 | 132.62 | 10 |
| $Pd_3$Au-Dap NPs | Palladium-gold bimetallic nanoparticle | 14.12 | 34.25 | 11 |
| MgCaFe-LDH@Au | Gold nanoparticles dispersed on magnesium–calcium–iron layered double hydroxide nanosheet | 29.69 | 71.9 | 12 |
| PCN | Oligopeptide-protected ultrasmall gold nanocluster | 1069 | NA | 13 |

| | | | | |
|---|---|---|---|---|
| β-CD-CuNCs | 6-thio-β-cyclodextrin-coated copper nanocluster | 32.87 | 0.45 | 14 |
| HP-$Pd_mRu_n$ NPs | Palladium@ruthenium nanozymes | 13.89 | 57.2 | 15 |
| $Pd_{91}$-GBLP | Palladium nanoparticles | 10.75 | 4.93 | 16 |
| Pt/H-TiN&SRF | Sorafenib-loaded titanium nitride and platinum nanoparticles | 32.03 | 11.39 | 17 |
| FA-$NH_2$-UiO-66-Cu | Copper single-atom-based metal-organic framework | 34.75 | 3.04 | 18 |

## References


1. Josephy, P. D.; Eling, T.; Mason, R. P., The horseradish peroxidase-catalyzed oxidation of 3,5,3',5'-tetramethylbenzidine. Free radical and charge-transfer complex intermediates. *Journal of Biological Chemistry* **1982,** *257* (7), 3669-3675.

2. Mvango, S.; Mashazi, P., Synthesis, characterization of copper oxide-gold nanoalloys and their peroxidase-like activity towards colorimetric detection of hydrogen peroxide and glucose. *Materials Science and Engineering: C* **2019,** *96*, 814-823.

3. Borah, P.; Baruah, D. J.; Mridha, P.; Duarah, R.; Baishya, R.; Das, M. R., In Situ Synthesis of Gold Nanoparticle-Decorated Borophene (Au@BNSs) Nanozymes with Glucose Oxidase and Peroxidase Activity for Colorimetric Detection of Glucose. *ACS Omega* **2025,** *10* (29), 32269-32281.

4. Ray, S.; Biswas, R.; Banerjee, R.; Biswas, P., A gold nanoparticle-intercalated mesoporous silica-based nanozyme for the selective colorimetric detection of dopamine. *Nanoscale Advances* **2020,** *2* (2), 734-745.

5. Tao, Y.; Ju, E.; Ren, J.; Qu, X., Bifunctionalized Mesoporous Silica-Supported Gold Nanoparticles: Intrinsic Oxidase and Peroxidase Catalytic Activities for Antibacterial Applications. *Advanced Materials* **2015,** *27* (6), 1097-1104.

6. Adeniyi, O.; Sicwetsha, S.; Mashazi, P., Nanomagnet-Silica Nanoparticles Decorated with Au@Pd for Enhanced Peroxidase-Like Activity and Colorimetric Glucose Sensing. *ACS Applied Materials & Interfaces* **2020,** *12* (2), 1973-1987.

7. Masud, M. K.; Yadav, S.; Islam, M. N.; Nguyen, N.-T.; Salomon, C.; Kline, R.; Alamri, H. R.; Alothman, Z. A.; Yamauchi, Y.; Hossain, M. S. A.; Shiddiky, M. J. A., Gold-Loaded Nanoporous Ferric Oxide Nanocubes with Peroxidase-Mimicking Activity for Electrocatalytic and Colorimetric Detection of Autoantibody. *Analytical Chemistry* **2017,** *89* (20), 11005-11013.

8. Liao, H.; Liu, G.; Liu, Y.; Li, R.; Fu, W.; Hu, L., Aggregation-induced accelerating peroxidase-like activity of gold nanoclusters and their applications for colorimetric Pb2+ detection. *Chemical Communications* **2017,** *53* (73), 10160-10163.

9. Cai, S.; Fu, Z.; Xiao, W.; Xiong, Y.; Wang, C.; Yang, R., Zero-Dimensional/Two-Dimensional AuxPd100–x Nanocomposites with Enhanced Nanozyme Catalysis for Sensitive Glucose Detection. *ACS Applied Materials & Interfaces* **2020,** *12* (10), 11616-11624.

10. Fu, Z.; Zeng, W.; Cai, S.; Li, H.; Ding, J.; Wang, C.; Chen, Y.; Han, N.; Yang, R., Porous Au@Pt nanoparticles with superior peroxidase-like activity for colorimetric detection of spike protein of SARS-CoV-2. *J Colloid Interface Sci* **2021,** *604*, 113-121.

11. Zhang, T.; Li, S.; Li, R.; Zhou, L.; Pei, P.; Zhao, H.; Wang, L.; Xie, D., Palladium-gold bimetallic nanoparticles stabilized by daptomycin for sensitive colorimetric detection of sulfide ions. *Analytica Chimica Acta* **2025,** *1348*, 343795.

12. Zheng, B.; Zhang, H.; Yu, G.; Geng, R.; Sang, Y.; Huang, G.; Wang, X.; Wang, J.; Hu, Y.; Ma, X., Cascade Catalytic Nanozymes Induce Tumor Ca2+ Overload and Ferroptosis by Reducing Energy Supply and Amplifying Oxidative Stress. *ACS Applied Materials & Interfaces* **2025,** *17* (23), 33498-33512.

13. Fan, D.; Ou, J.; Chen, L.; Zhang, L.; Zheng, Z.; Yu, H.; Meng, X.; Zhu, M., An Oligopeptide-Protected Ultrasmall Gold Nanocluster with Peroxidase-Mimicking and Cellular-Imaging Capacities. *Molecules* **2022,** *28* (1).

14. Zhong, Y.; Deng, C.; He, Y.; Ge, Y.; Song, G., Exploring a monothiolated β-cyclodextrin as the template to synthesize copper nanoclusters with exceptionally increased peroxidase-like activity. *Microchimica Acta* **2016,** *183* (10), 2823-2830.

15. Wang, J.; Zeng, Q.; Cao, K.; Cui, Y.; Xiao, H.; Wang, L., Green synthesis of hawthorn polysaccharide-stabilized Pd@Ru nanozymes for sensitive colorimetric detection of organophosphorus pesticides. *International Journal of Biological Macromolecules* **2025,** *329*, 147780.

16. Cui, Y.; Lai, X.; Liu, K.; Liang, B.; Ma, G.; Wang, L., Ginkgo biloba leaf polysaccharide stabilized palladium nanoparticles with enhanced peroxidase-like property for the colorimetric detection of glucose. *RSC Adv* **2020,** *10* (12), 7012-7018.

17. Liu, J.; Dong, S.; Gai, S.; Li, S.; Dong, Y.; Yu, C.; He, F.; Yang, P., Four Birds with One Stone: A Bandgap-Regulated Multifunctional Schottky Heterojunction for Robust Synergistic Antitumor Therapy upon Endo-/Exogenous Stimuli. *ACS Nano* **2024,** *18* (34), 23579-23598.

18. Wang, H.; Zhang, Z.; Wang, X.; Jin, X.; Gao, X.; Yu, L.; Han, Q.; Wang, Z.; Song, J., Copper Single-Atom-Based Metal–Organic Framework for Ultrasound-Enhanced Nanocatalytic Therapy. *Nano Letters* **2024,** *24* (31), 9700-9710.
